# Music Intervention Leads to Increased Insular Connectivity and Improved Clinical Symptoms in Schizophrenia

**DOI:** 10.3389/fnins.2017.00744

**Published:** 2018-01-23

**Authors:** Hui He, Mi Yang, Mingjun Duan, Xi Chen, Yongxiu Lai, Yang Xia, Junming Shao, Bharat B. Biswal, Cheng Luo, Dezhong Yao

**Affiliations:** ^1^The Clinical Hospital of Chengdu Brain Science Institute, MOE Key Lab for Neuroinformation, University of Electronic Science and Technology of China, Chengdu, China; ^2^The Four People's Hospital of Chengdu, Chengdu, China

**Keywords:** schizophrenia, music intervention, resting-state fMRI, functional connectivity, insular cortex, validation analysis

## Abstract

Schizophrenia is a syndrome that is typically accompanied by delusions and hallucinations that might be associated with insular pathology. Music intervention, as a complementary therapy, is commonly used to improve psychiatric symptoms in the maintenance stage of schizophrenia. In this study, we employed a longitudinal design to assess the effects of listening to Mozart music on the insular functional connectivity (FC) in patients with schizophrenia. Thirty-six schizophrenia patients were randomly divided into two equal groups as follows: the music intervention (MTSZ) group, which received a 1-month music intervention series combined with antipsychotic drugs, and the no-music intervention (UMTSZ) group, which was treated solely with antipsychotic drugs. Resting-state functional magnetic resonance imaging (fMRI) scans were performed at the following three timepoints: baseline, 1 month after baseline and 6 months after baseline. Nineteen healthy participants were recruited as controls. An FC analysis seeded in the insular subregions and machine learning techniques were used to examine intervention-related changes. After 1 month of listening to Mozart music, the MTSZ showed increased FC in the dorsal anterior insula (dAI) and posterior insular (PI) networks, including the dAI-ACC, PI-pre/postcentral cortices, and PI-ACC connectivity. However, these enhanced FCs had vanished in follow-up visits after 6 months. Additionally, a support vector regression on the FC of the dAI-ACC at baseline yielded a significant prediction of relative symptom remission in response to music intervention. Furthermore, the validation analyses revealed that 1 month of music intervention could facilitate improvement of the insular FC in schizophrenia. Together, these findings revealed that the insular cortex could potentially be an important region in music intervention for patients with schizophrenia, thus improving the patients' psychiatric symptoms through normalizing the salience and sensorimotor networks.

## Introduction

Neuropsychiatric conditions, such as schizophrenia, display a complex and diverse neurobiology, which has long been associated with difficulty in distinguishing between the “self” and “non-self,” as well as with an uncertainty regarding whether one's actions and thoughts are independent from external influences. Various passivity symptoms, such as auditory verbal hallucinations, thought insertion, and emotion processing, especially in response to emotional stimuli, may be caused by these experiences. These symptoms are often referred to as first-rank symptoms, which play a key role in the diagnosis of patients with schizophrenia (Waters and Badcock, 2010). Neuroimaging data also support the idea that these symptoms are related to altered brain functional connectivity (FC) (Fornito et al., 2012; Wojtalik et al., 2017). Prolonged treatment with antipsychotic drugs is a common choice for the remission stage of schizophrenia. Complementary therapies, such as music intervention (Talwar et al., 2006) and cognitive-behavioral intervention (Rector and Beck, 2001), are also used for patients with schizophrenia. Specifically, recent studies of patients with schizophrenia showed that music intervention could significantly improve psychiatric symptoms (Mössler et al., 2011; Lu et al., 2013).

Music is one of the oldest and most basic sociocognitive domains of the human species. It is considered as a profound capacity to bind individuals together emotionally and to change our physiological behavior, emotions, and subjective perception of time (Habibi and Damasio, 2014). Music listening and music therapy (such as music performance and/or listening by patients, etc.) are often justified by the proposed need for a medium for communication and expression. Listening to Mozart K.448 could temporarily enhance spatial-temporal reasoning on humans (Rauscher et al., 1993). Other forms of music were found to be equally effective in the short-term (Gardiner et al., 1996; Rauscher et al., 1997). The discovery of these effects of music opened a new page for the study of the impact of music on humans. Furthermore, music therapy is one therapeutic method that uses musical experiences to help people with serious mental disorders develop relationships and address issues that they may not be able to by using words alone (Bruscia, 1998; Gold et al., 2009). Research has extensively and continuously examined the cognitive effects of music listening on listeners and music therapy on patients with dementia, anxiety, and schizophrenia (Pavlicevic et al., 1994; Foster and Valentine, 2001; Chan et al., 2003).

The effect of music intervention is associated with the regulation of behaviors, such as emotion and sensorimotor processing in our bodies (Damasio, 2001; Habibi and Damasio, 2014; Luo et al., 2014; Li et al., 2015) that are considered core fields of abnormality in schizophrenia (Wylie and Tregellas, 2010). To assess the effect of music therapy on patients with schizophrenia, almost all studies have compared both individual and groups of patients receiving standard care with or without music therapy (Talwar et al., 2006; Ulrich et al., 2007). The duration of these studies varied from 1 to 4 months (40–60 min per week), while no later follow-up assessments over a longer term were included. More practically, clinical reports have suggested that music therapy can have uniquely motivating, emotionally expressive and relationship-building qualities in schizophrenia (Rolvsjord, 2001; Solli, 2008). Music therapy has been shown to significantly increase the patients' level of interest in external events and to diminish their negative symptoms (Tang et al., 1994). In addition, music listening has also been performed at the group level in patients. After music intervention, significant advantages were detected in some measures concerning personal relations and subjectivity in patients with schizophrenia (Hayashi et al., 2002). Cognitive task performance could also be facilitated after listening to music by Mozart in patients with schizophrenia (Glicksohn and Cohen, 2000). However, the particular mechanism behind this phenomenon is still poorly understood; the neural system changes induced by the long-term effects of music intervention could translate into clinically meaningful effects. Importantly, neuroimaging studies have highlighted that music can modulate the state of neural systems, e.g., the insular network (Baumgartner et al., 2006; Koelsch et al., 2006). Music training could lead to significant reorganization in insula-based networks, potentially facilitating the high-level cognitive and affective functions that are associated with the integration of multisensory information in the context of music performance (Zamorano et al., 2017). In addition, the right anterior insula is also a key node in the brain's singing network, which is responsible for the integration of salient signals across multiple sensory and cognitive domains that guides vocal behavior (Kleber et al., 2017). Thus, the insula might be an important region related to music intervention in patients with schizophrenia.

The human insular cortex forms a distinct lobe and involves three major functionally unique subregions (Deen et al., 2011), including the ventral anterior insula (vAI), dorsal anterior insula (dAI), and the posterior insula (PI). The dAI functions as an integral hub in integrating the interactions between other brain networks involved in externally oriented attention and self-related cognition (Kurth et al., 2010; Uddin et al., 2014), the vAI appears to be more involved in affective processes, and the PI is associated with sensorimotor processing (Chang et al., 2013). Of these three subregions, particular attention has been paid to the dAI because it is a key node in the salience network (SN), which contributes to stimulus detection and salience processing (Kurth et al., 2010; Uddin et al., 2014; Nomi et al., 2016). Moreover, task-based investigations have revealed that the dAI is the most flexible insula subregion (Yeo et al., 2014). The dAI has also been reported to facilitate the detection of salient exogenous stimuli and to coordinate network switching between the default mode and central executive networks (Menon and Uddin, 2010). Thus, the insula provides the basis for a sense of the physiological condition of the body (sensorimotor processing) and for the representations of signals from the external environment (external emotional stimuli) (Craig, 2004; Singer et al., 2009). In other words, the insula is a likely candidate region for where the integration of internal and external information and the maintenance of the balance between them occur.

Many deficits observed in schizophrenia might be related to insula pathology (Wylie and Tregellas, 2010; Dong et al., 2017). Dysfunction of the insula may contribute to the difficulty in recognizing emotional facial expressions in patients with schizophrenia (Williams et al., 2007), as well as to the difficulties in evaluating and creating emotionally vocal expressions (Mitchell et al., 2004). Individuals with schizophrenia have an impaired right anterior insula modulation of the central executive and default mode networks (Jiang et al., 2017), which could predict a patient's cognitive performance (Moran et al., 2013). Furthermore, the loss of insight that is seen in schizophrenia has also been associated with morphometry of the posterior insula (Palaniyappan et al., 2011). In response to a painful stimulus, lower activation of the middle-posterior insula provided support for the existence of a basic deficit in interoceptive perception in schizophrenia (Linnman et al., 2013). Importantly, the insula, especially the anterior part, is involved in identifying subjectively self-generated from externally generated information. According to one interpretative hypothesis (Wylie and Tregellas, 2010), the abnormalities in the insular cortex might lead to the attribution of internally generated sensory information to an external source, thus eventually contributing to the hallucinations in patients with schizophrenia.

Therefore, in this study, we investigated whether the effects of long-term music intervention in patients with schizophrenia could positively improve patients' symptoms and behaviors through changes in the insula functional network. Based on evidence from the diagnosis of patients with schizophrenia and from functional neuroimaging studies, we hypothesized that music intervention could positively modulate the FC of the insula in patients with schizophrenia. These intervention-affected areas are important for regulation and self-reflection. These altered FCs could positively improve patients' symptoms and behaviors. To validate our hypothesis, we used resting-state functional magnetic resonance imaging (fMRI) and machine learning approaches to examine the effects of long-term music intervention on patients with schizophrenia.

## Materials and methods

### Subjects

Seventy-five subjects (56 patients and 19 healthy controls) participated in this study. The patients with schizophrenia were recruited from the clinical hospital of Chengdu Brain Science Institute (CBSI). The inclusion criteria for the inpatients in this study was a primary diagnosis of schizophrenia, according to the Structured Clinical Interview for the DSM-IV Axis I disorders-clinical version (SCID-I-CV), by two experienced psychiatrists. The exclusion criteria were acute psychotic symptoms, a secondary diagnosis of organic psychosis or dementia, unstable drug treatment, not being able to mingle in a group, as well as the presence of other arts interventions (art, dance, and movement). A set of matched MRI data of 19 healthy controls (HCs), which was used as a healthy reference, was also obtained from the clinical hospital of CBSI research databases. The HCs were screened for a history of medical or neuropsychiatric illness, as well as for major neurological or psychiatric illness in their first-degree relatives. The controls and patients were matched for age, gender and years of education.

### Design

**Figure 1:**
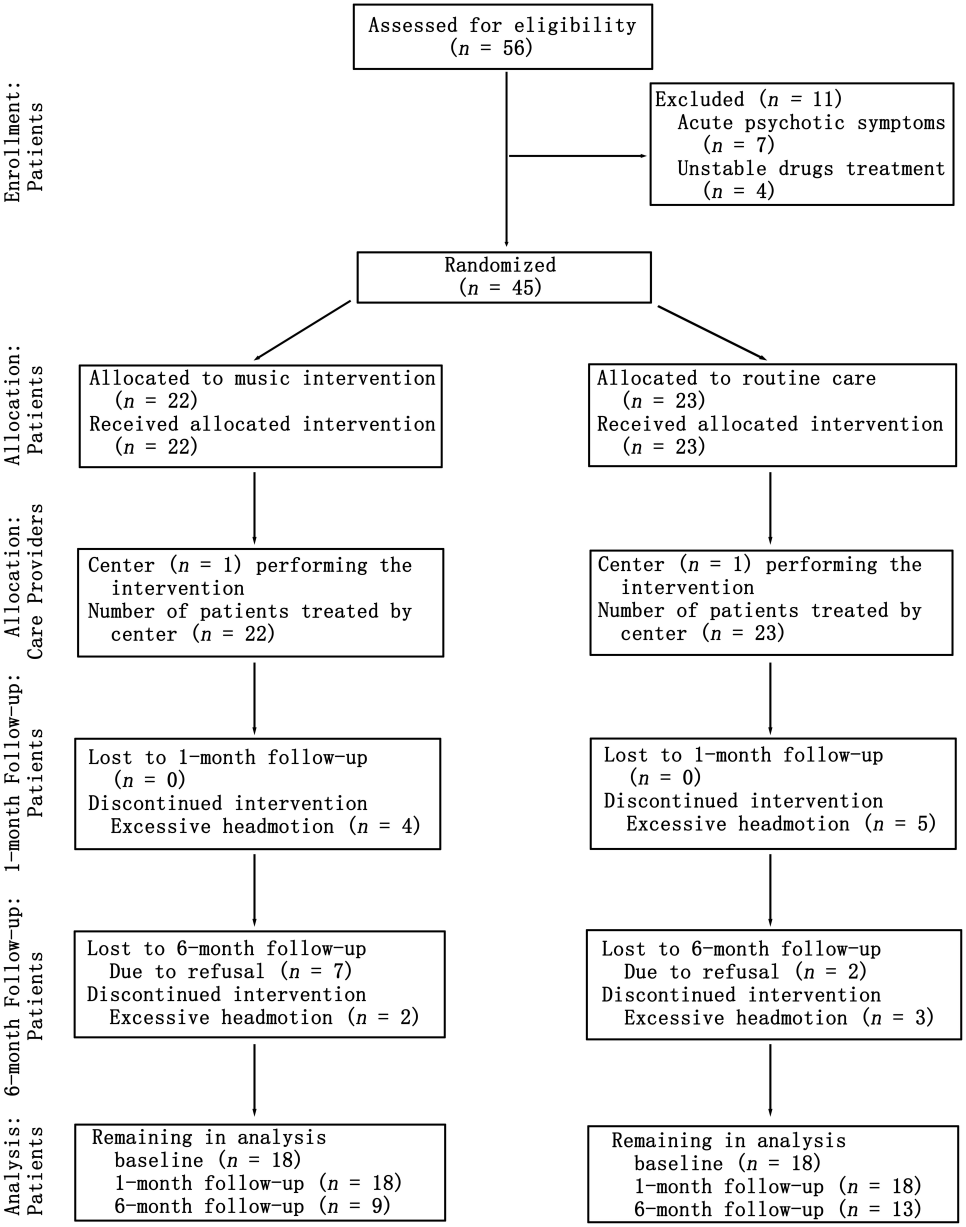
Consort diagram showing the flow of patients through the study from baseline to the 1- and 6-month follow-ups.

To examine the effect of music intervention sessions on patients with schizophrenia, we conducted a quasi-randomized controlled trial. The study spanned a period of 6 months. The design consisted of three timepoint-tests, including a baseline test, a 1 month later test and a 6 months later test, that were used in this study. The experimental inpatients and controlled inpatients were compared using the reference of the HCs. The inpatients with schizophrenia were randomly divided into two groups by psychiatrists, a music intervention schizophrenia group (MTSZ, 22 patients) and a no-music intervention schizophrenia (UMTSZ, 23 patients), that only controlled for the balance of age, gender and education level in the two patient groups. The experimental patient group underwent music intervention in addition to a stable drug treatment strategy (changeless antipsychotic drugs and their doses). The control group received the stable drug treatment as well. The medication dosage information of the patients is shown in Table S1. The blinded assignment and assessments, which included the resting-state MRI, psychiatric symptoms and neuropsychological measurements, were performed at three timepoints during the music intervention in the two patient groups. A flow chart of the patient interventions throughout the study is shown in Figure 1. The patients with schizophrenia were given information about the study procedures and the music intervention. Every subject provided written consent to participate in this study. Part of consent include the exact information of the music intervention processing and security provisions. The study was approved by the Ethics Committee of the clinical hospital of CBSI in accordance with the Helsinki Declaration. All the methods were carried out in accordance with the approved guidelines.

### Content of music intervention

In this study, the musical piece used was Mozart's sonata K.448, which has been widely used in scientific studies to assess the effects of music (Rauscher et al., 1993; Coppola et al., 2015; Xing et al., 2016). One professional music therapist, who had long-term working experience in music therapy, participated in this experiment. The MTSZ group received a 1-month course in music intervention (Mozart's sonata K.448 music listening, 30 sessions), in addition to antipsychotic drugs. Each session took 30 min per day. During these sessions, the main activity that the patients participated in was the peaceful listening of the music of Mozart's sonata K.448 from a stereo system that was installed in a quiet room. At the beginning of the experiment, the therapist introduced the background of music to the patients. Finally, after each session, the therapist recorded the information about the musical experience of the patients. The UMTSZ was set as the control group that was treated solely with antipsychotic drugs.

### Psychiatric and neuropsychological assessment

Because the patients stayed in different wards, three evaluators of the neuropsychological assessments, and one psychiatrist assessed them. The psychiatric symptoms of the patients were assessed by a psychiatrist using the Positive and Negative Symptom Scale (PANSS). For the neuropsychological assessment, before the start of the study, the evaluators were trained in the use of observer instruments to achieve a high inter-rater reliability. Then, we administered the Block Design Test (BDT) and the Benton Visual Retention Test (BVRT), as well as the Spatial Maze Test from the Wechsler Adult Intelligence Scale Revised (WAIS-R) (Wechsler, 1981) to the two patient groups. The BDT, which reflects visuospatial ability (Kaufman, 2001), requires the subjects to duplicate 10 target patterns using a set of two-colored blocks. The patterns were presented in order of ascending difficulty. The BVRT is a well-established neurodiagnostic instrument that has been used to assess visuospatial perception and retention (Tamkin and Kunce, 1985). The target patterns contained geometric and abstract figures and were displayed to the subjects for 10 s. After that, the subjects were required to duplicate the figures from immediate memory. Finally, the Spatial Maze task, which also comes from the WAIS-R, is one of the most reliable measures of visuospatial anterograde memory function.

### MRI data acquisition and preprocessing

The experiments were performed on a 3T MRI scanner (GE DISCOVERY MR750). at the University of Electronic Science and Technology of China. During scanning, we used foam padding and ear plugs to reduce head motion and scanning noise, respectively. The resting-state functional MRI data were acquired using gradient-echo echo planar imaging sequences (repetition time [TR] = 2,000 ms, echo time [TE] = 30 ms, flip angle [FA] = 90°, matrix = 64 × 64, field of view [FOV] = 24 × 24 cm^2^, slice thickness/gap = 4 mm/0.4 mm), with an eight channel-phased array head coil. All subjects underwent a 510-s resting state scan to yield 255 volumes (32 slices per volume). The first five volumes were discarded for the magnetization equilibrium. Subsequently, high-resolution T1-weighted images were acquired using a 3-dimensional fast spoiled gradient echo sequence (TR = 6.008 ms, FA = 9 degree, matrix = 256 × 256, FOV = 25.6 × 25.6 cm^2^, slice thickness = 1 mm, no gap, 152 slices). During the resting-state fMRI, all the subjects were instructed to have their eyes-closed and to move as little as possible without falling asleep.

The functional data preprocessing was performed using SPM8 (Statistical Parametric Mapping, http://www.fil.ion.ucl.ac.uk/spm/). A series of preprocessing steps were performed for each subject as follows: (1) slice timing correction; (2) head motion correction; (3) normalization: the functional data were spatially normalized (3 ^*^ 3 ^*^ 3 mm) to the EPI template; (4) the images were smoothed by a 6-mm full width at half maximum Gaussian kernel; (5) temporal filtering was performed at bandpass 0.01–0.08 Hz; and (6) nuisance signals were regressed out, including white matter, cerebrospinal fluid, and 12 motion parameters (x-,y-,z-translations, three rotations, their derivatives), except for the global signal due to a recent excellent study that demonstrated that altered global brain signal was observed in patients with schizophrenia, which may underlie profound alterations in the neural information flow in patients with schizophrenia (Yang et al., 2014). In addition, a recent study also demonstrated that head motion has a substantial impact on FC (Power et al., 2012). Thus, any subjects who had a maximum translation in any of the cardinal directions larger than 2.0 mm or a maximum rotation larger than 2.0 degree were excluded from subsequent analysis. In addition, framewise displacement (FD) was evaluated in the three groups as suggested by Power et al. (2012).

The structural images were processed using the SPM8 toolbox, with spatial normalization to the MNI-space using a diffeomorphic anatomical registration through exponentiated lie algebra (DARTEL), and segmentation into gray matter (GM), white matter and cerebrospinal fluid. The segmented GM was modulated using nonlinear deformation. Then, we obtained the total GM volume and total intracranial volume (TIV) of each subject. The GM volumes of all the subjects were normalized by dividing the individual TIV score of each subject. The GM value is related to the local functional connectivity strength across a brain region (Liang et al., 2013). Thus, to avoid the effects of GM, the patients and HCs' normalized GM volumes at baseline were entered as the global variable to correct for the global GM volume effect across subjects in the statistical analysis.

### Functional connectivity mapping

The same analysis was performed in all three groups. In the previous literature, three subregions of the insula, including the ventral anterior insula (vAI), dorsal anterior insula (dAI) and the posterior insula (PI), were subdivided based on the clustering of the FC patterns in the unilateral insula (Deen et al., 2011). According to the template of the insula from the findings of Deen et al. each ROI was used as a seed in the whole-brain FC analysis in each of the three groups. The mean BOLD series of each ROI were extracted from these seeds. Subsequently, an FC analysis was performed between the seed and all the voxels in the brain. The resulting correlation coefficients were transformed using Fisher's r-to-z transformation.

### Statistical analysis

#### Baseline functional connectivity analysis between the patients and HCs

We established baseline abnormalities between the healthy controls and the patients with schizophrenia through a voxel wise two-sample *t*-test, with gender, years of education, and age as covariates, within an explicit mask from the union set of the one-sample *t*-test results of the two groups. Due to the greater number of patients than the number of healthy controls, an equal number of the HC group and patients with schizophrenia who were randomly selected from the two patient groups was entered into the statistical comparison. To ensure a high reproducibility of our results at baseline, these steps were repeatedly performed 200 times, which led to a total of 200 two-sample *t*-test results. For each insular subregion, we calculated the probability maps of the comparison results, where the voxels exhibited significant group differences (*P* < 0.05, cluster level false discovery rate corrected) across the total 200 tests.

#### Longitudinal analysis in patients

After tests for normality, homogeneity of variance and Mauchly's test of sphericity through Matlab 2016a software, the repeated measures ANOVA and *post-hoc* analyses were performed to assess the music intervention ^*^ time interaction and the main effects of music intervention and time on the FC, as well as on the symptom and neuropsychological measurements. Age, gender, illness duration, education characteristics, GM, and medication dosage were used as the potential confounding covariates in the statistical analysis. Specifically, in each FC map, the within-group *z*-values map was analyzed using a one-sample *t*-test (*P* < 0.05, cluster level false discovery rate corrected). Then, we restricted the ANOVA to the mask of the union of the one-sample *t*-test results. The significance threshold of the group differences for the ANOVA was set to *P* < 0.05 (cluster level false discovery rate corrected; Forman et al., 1995). We extracted the FC, symptom and neuropsychological measurements that showed significant changes in the repeated measures ANOVA from the MTSZ and UMTSZ groups for the *post-hoc* analysis.

### Validation analyses

Validation analyses were included to address the potential effects related to music intervention in more detail. Two established classifiers [support vector machine (SVM) and *post-hoc* analysis(PHA)], in which two levels were set, including a level with an intervention effect and a level without an intervention effect, were applied to the intra and inter group validation analyses. To identify the difference between the groups, two classification models (classifiers_MTSZ and classifiers_UMTSZ) were conducted, respectively. First, in the intra-group validation analysis, we used the leave one out cross validation (LOOCV) strategy to predict the intervention effect in the MTSZ and UMTSZ groups. Second, for the inter-group validation analysis, two classification models were built based on the data of the MUSZ and UMTSZ groups. Then, two decision models were used to predict the intervention effect in the validation groups; the other patient and HC groups, in which the baseline data of the patients would be classified as a level without an intervention effect, 1-month data of the patients and HCs would be predicted to divide into a level with an intervention effect. The validation performances of the two classification models were adopted to evaluate the therapeutic effect. In other words, a higher performance of the model was reflective of a better therapeutic effect of these classifiers. Detailed information can be found in section 3 of the Supplemental Information.

Furthermore, to predict the changes in the individual PANSS scores and neuropsychological measurements in the MTSZ group, a support vector regression (SVR) algorithm was used to calculate the regression model, in which the percentage of change scores (1 – [score at 1-month/score at baseline]) were estimated based on the FCs at baseline. An epsilon SVR based on the model implemented in the SVM (Chang and Lin, 2011) with a linear kernel and default parameters was applied, using a LOOCV procedure.

## Results

Forty-five patients with schizophrenia finished the randomized controlled trial at two time-points (baseline and 1-month follow-up). Nine of them (four in MTSZ and five in UMTSZ) were excluded due to the excessive head motion during the MRI scans. Thus, 18 MTSZ, 18 UMTSZ, and 19 HC were included in the following analysis. The demographic information is displayed in Table 1.

**Table 1:** Participant fundamental information.

	**MTSZ patients baseline**	**UMTSZ patients baseline**	**MTSZ patients 1-month**	**UMTSZ patients 1-month**	**Healthy controls**	***p***
Gender (Male/Female)	5/13	5/13	–	–	7/12	0.787^a^
Age (years)	45.38 ± 9.69	45.72 ± 7.63	–	–	44.42 ± 4.70	0.863^b^
Education level (years)	11.94 ± 3.24	11.22 ± 2.90	–	–	11.36 ± 2.81	0.641^b^
Head motion	0.062 ± 0.031	0.089 ± 0.065	0.078 ± 0.046	0.088 ± 0.067	0.075 ± 0.055	0.560^b^
Duration of illness (years)	19.66 ± 11.11	18.00 ± 8.18	–	–		0.611^c^
Medication dosage in CPZ equivalents (mg)	339.23 ± 94.15	320.53 ± 142.50	–	–		0.645^c^

*MTSZ, Music intervention schizophrenia; UMTSZ, no-music intervention schizophrenia; CPZ, chlorpromazine; Indicated values are shown mean ± standard deviation*.

a *Indicates the p-values for the comparisons (Chi-square test) among the MTSZ(baseline), UMTSZ(baseline), and healthy controls*.

b *Indicates the p-values for the comparisons (Analysis of variance) among the MTSZ(baseline), UMTSZ(baseline), and healthy controls*.

c *Indicates the p-values for the comparisons (Two-sample t-tests) between the MTSZ and the UMTSZ at baseline*.

To further examine the effect of music intervention on the insular networks, we did the follow-up visits 6 months later, as we expected that the effect of music intervention would vanish gradually after the period of intervention (Guétin et al., 2009). However, nine patients with schizophrenia (25%) were not recalled because they were no longer interested in the experiment after being discharged from the hospital. Five patients with schizophrenia with excessive head motion were also excluded, so thirteen (13/18) UMTSZ and nine (9/18) MTSZ patients were included in the follow-up analysis. Through comparison analysis, the long-term effect of music intervention was evaluated.

### Changes in clinical and neuropsychological measurements

Through a repeated measures ANOVAs analysis, we observed significant music intervention main effects and music intervention ^*^ time interaction in the PANSS and BVRT scores (Table 2). None of the symptoms or neuropsychological measurements showed significant main effects of time. *Post-hoc* analysis revealed no significant difference between the MTSZ and UMTSZ groups at baseline for any of the features of the psychiatric symptoms or neuropsychology. The significant increase in the BVRT and the decrease in the PANSS scores were found in the MTSZ group following music intervention, but not in the UMTSZ group (Table 2). Furthermore, the effects of music intervention had vanished in the MTSZ group at the follow-up visits after 6 months [comparison results between 1 and 6 months from the baseline: BVRT: *t*_(17)_ = 3.76, *p* = 0.001; PANSS-total score: *t*_(17)_ = 5.24, *p* < 0.001; PANSS-positive score: *t*_(17)_ = 2.51, *p* = 0.022; PANSS-negative score: *t*_(17)_ = 2.70, *p* = 0.015; PANSS- general score: *t*_(17)_ = 3.56, *p* = 0.002].

**Table 2:** The main effects of time and music intervention factor, as well as the music intervention ^*^ time interaction on PANSS and neuropsychological scores in patients through repeated measure ANOVA.

	**MTSZ**	**UMTSZ**	**MTSZ**	**UMTSZ**	**MTSZ**	**UMTSZ**	**Interaction effects**		**Music main effects**		***Post-hoc*** **(paired** ***t-*****test)**		***Post-hoc*** **(*****t-*****test2)**	
	**Baseline 18/18**	**Baseline 18/18**	**1-month 18/18**	**1-month 18/18**	**6-month 9/18**	**6-month 13/18**	***F*-value**	***P*-value**	***F*-value**	***P*-value**	***P*-value (MTSZ)**	***P*-valve (UMTSZ)**	***P*-value (baseline)**	***P*-valve (1 month)**
Block design test	23.83 ± 9.58	20.11 ± 10.89	25.5 ± 7.11	22.78 ± 10.00	22.21 ± 11.09	20.63 ± 9.04	0.186	0.669	3.487	0.071	0.32	0.12	0.284	0.272
Spatial maze	5.50 ± 1.10	4.72 ± 1.96	5.77 ± 1.06	4.83 ± 2.09	4.93 ± 3.51	3.73 ± 2.59	0.31	0.581	1.69	0.202	0.24	0.57	0.152	0.783
BVRT	3.28 ± 2.05	3.55 ± 2.48	4.33 ± 2.79	3.67 ± 2.43	2.69 ± 1.47	3.87 ± 3.43	5.842	0.021^*^	8.914	0.005^**^	0.004^**^	0.631	0.717	0.33
PANSS-total score	62.89 ± 17.41	64.11 ± 11.73	54.78 ± 14.56	63.50 ± 12.21	63.60 ± 15.23	63.40 ± 14.81	9.509	0.004^**^	12.861	0.001^**^	0.002^**^	0.513	0.806	0.059
PANSS-positive score	12.89 ± 4.38	10.67 ± 4.60	10.66 ± 3.3	10.61 ± 4.64	12.71 ± 5.64	10.71 ± 3.94	13.294	0.001^**^	14.693	0.001^**^	0.002^**^	0.331	0.147	0.967
PANSS-negative score	21.78 ± 9.24	23.39 ± 5.98	19.39 ± 8.88	23.11 ± 6.67	23.11 ± 7.63	21.91 ± 7.27	3.005	0.092	4.794	0.036^*^	0.017^*^	0.738	0.539	0.164
PANSS-general score	28.22 ± 7.03	30.05 ± 5.97	24.72 ± 5.00	29.78 ± 6.05	27.78 ± 6.15	30.78 ± 6.13	8.228	0.007^**^	11.311	0.002^**^	0.005^**^	0.311	0.405	0.010^*^

*Indicated values are shown mean ± standard deviation. BVRT, Benton visual retention test; PANSS, positive and negative symptom scale; “t-test2” means the t-test between MTSZ and UMTSZ*.

* *p < 0.05*,

** *p < 0.01*.

### Baseline functional connectivity abnormalities between patients and HC

**Figure 2:**
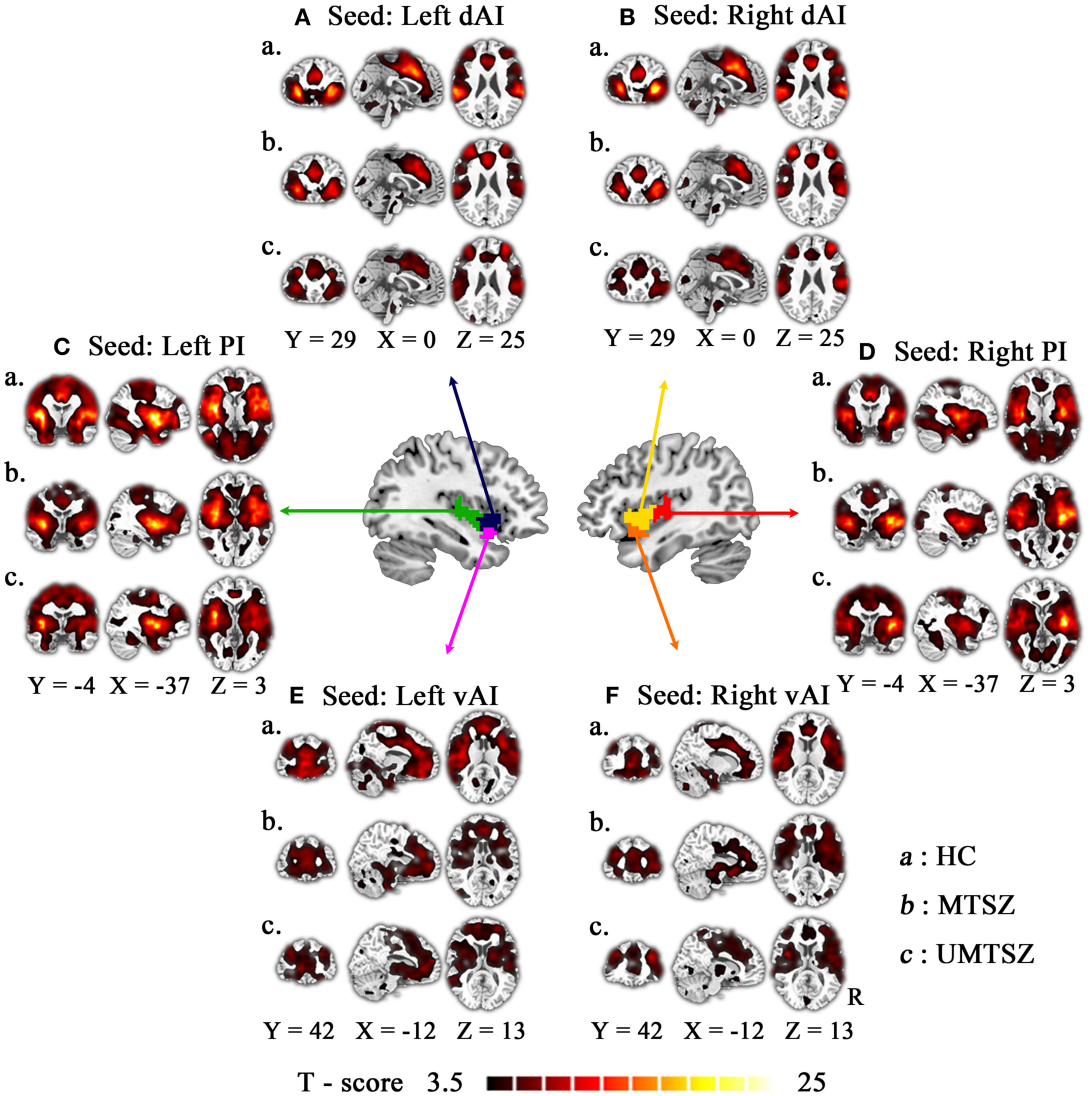
The FC patterns of each seed in three groups. Patterns of significant positive correlations with the following six seeds: bilateral dorsal anterior insula [**(A):** left dAI; **(B)**: right dAI], bilateral posterior insula [**(C)**: left PI; **(D)**: right PI], bilateral ventral anterior insula [**(E)**: left vAI; **(F)**: right vAI], in healthy control (“a”: HC) and music intervention patients with schizophrenia (“b”: MTSZ), and music no-intervention patients with schizophrenia (“c”: UMTSZ). For display purposes, all of the maps are shown with *t* score between 3.5 and 10.

**Figure 3:**
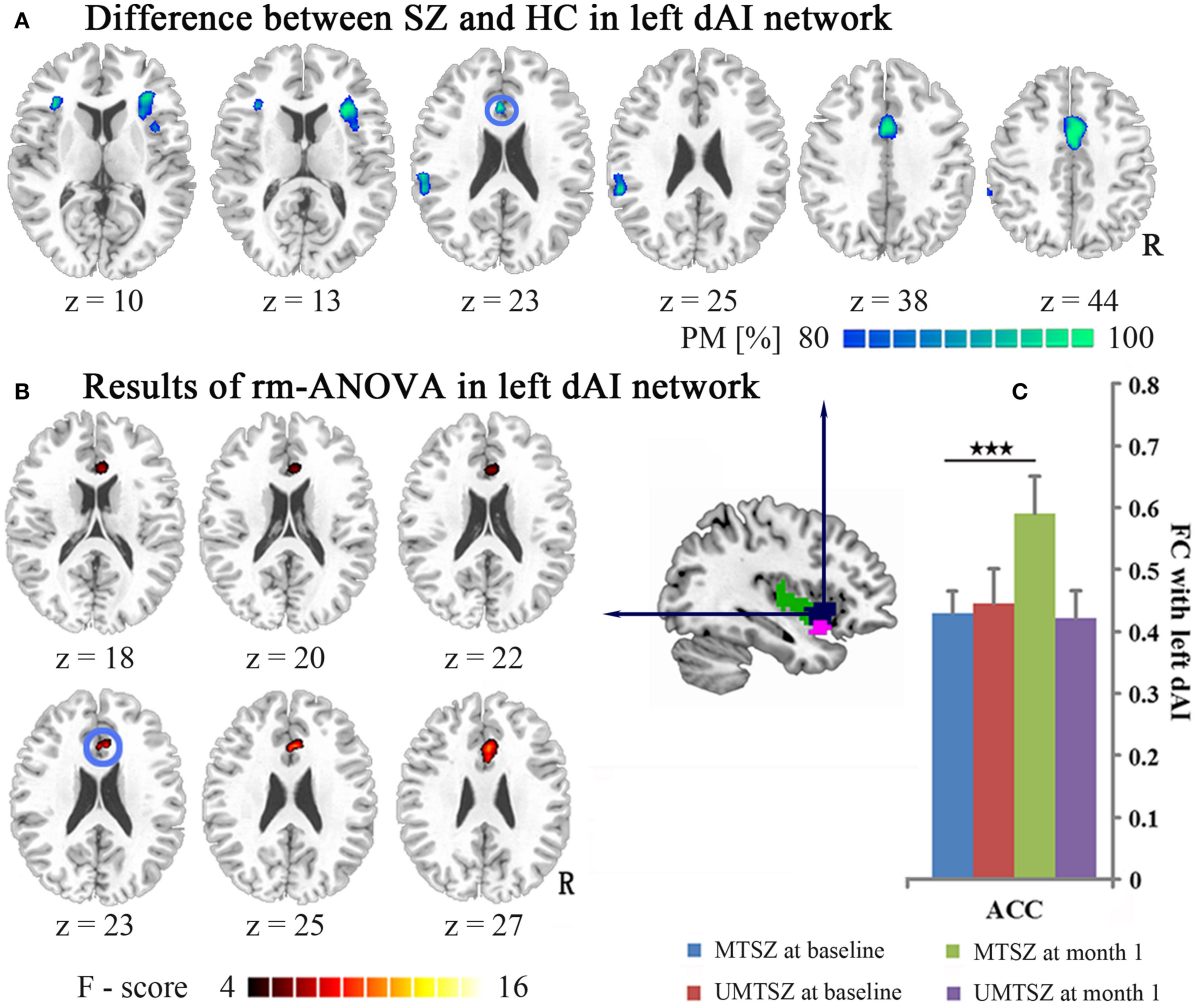
Music intervention ^*^ time interactions on the FC map of the left dAI. **(A)** Denotes the altered FC in patients compared with the HCs. The cool color indicates decreased FC. The maps are shown with probability scores between 80 and 100%. **(B)** Denotes the significant music intervention ^*^ time interaction that was observed between the left dAI and the ACC. **(C)** The bar maps present the FC differences between-group and within-group in the regions showing significant music intervention ^*^ time interaction. The data are expressed as the mean value + standard error. ^***^p < 0.001. PM: probability map. The blue circle marks the same region in **(A,B)**, which represents the positively modulated region through music intervention in patients.

**Figure 4:**
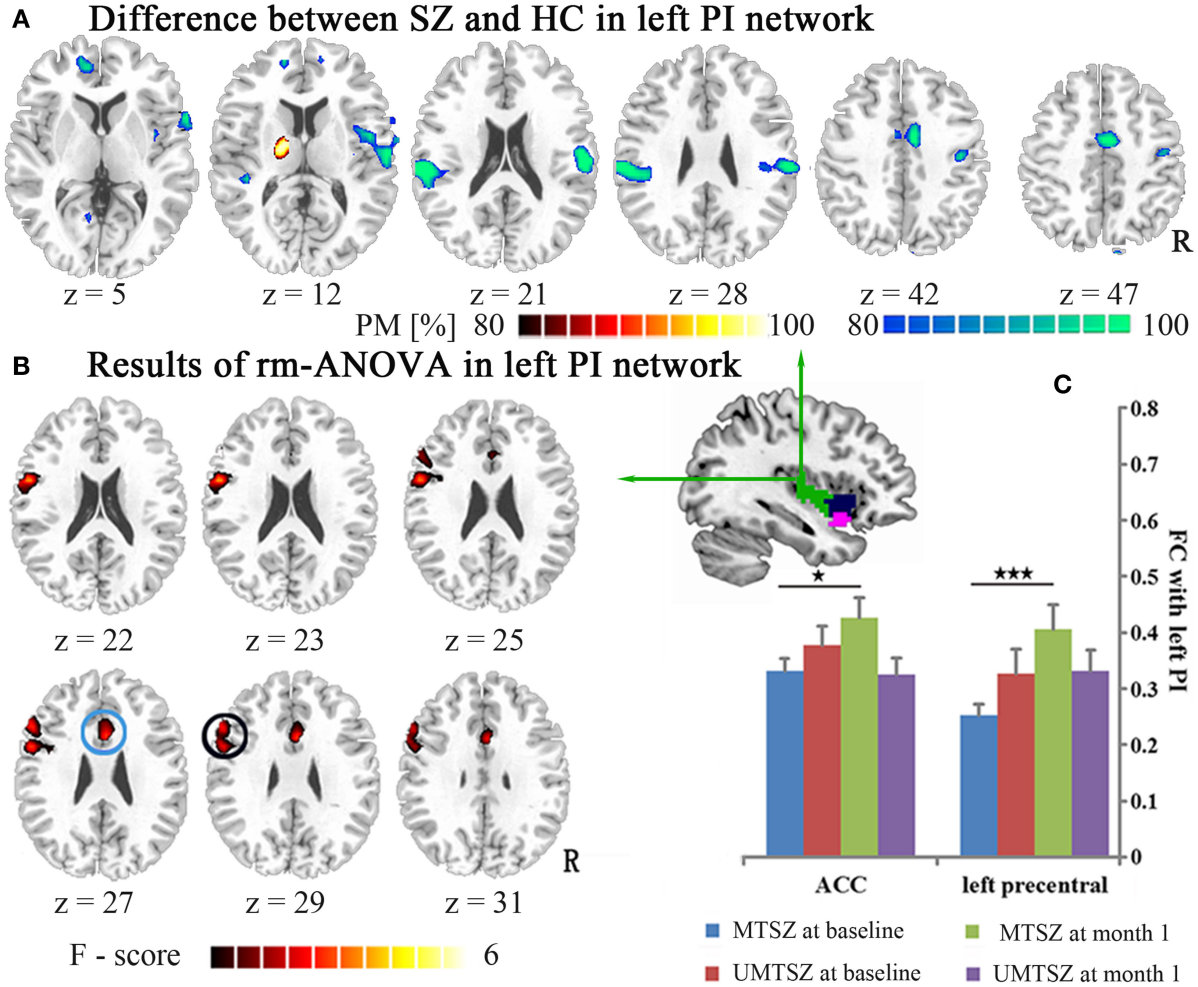
Music intervention ^*^ time interaction effect on the FC of the left PI. **(A)** Denotes the altered FC in the patients compared with the HCs. The cool color indicates decreased FC, and the hot color indicates increased FC. The maps are shown with probability score between 80 and 100%. **(B)** Denotes the significant music intervention ^*^ time interactions that were observed in the left PI-ACC FC and the PI-left precentral FC. **(C)** The bar maps present the FC differences between-group and within-group. The data are expressed as the mean value + standard error. ^*^p < 0.05, ^***^p < 0.001. PM: probability map. The circles mark in **(B)**, which represent the positively modulated region through music intervention in patients in **(C)** (blue circle: ACC; black circle: left precentral).

**Figure 5:**
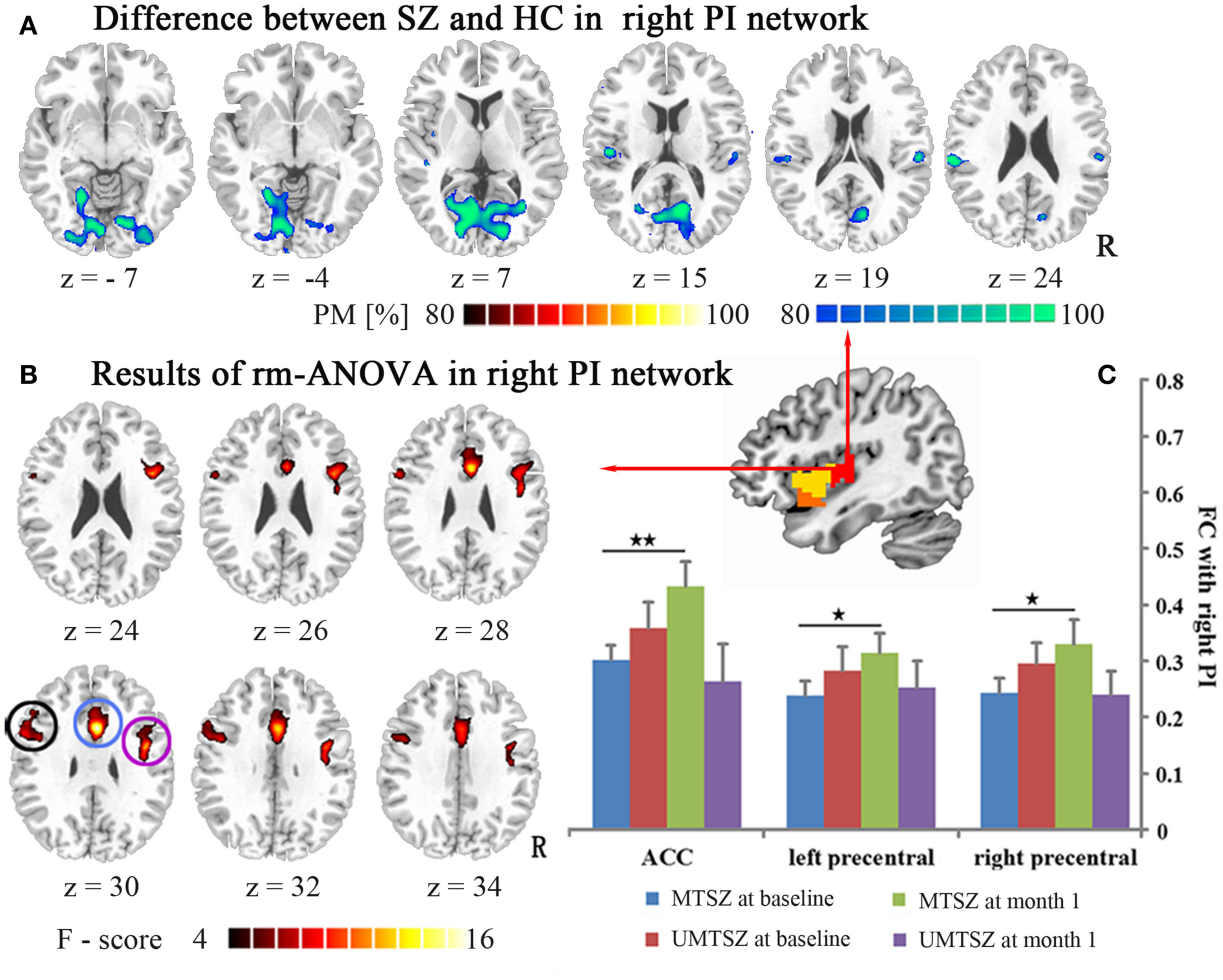
Music intervention ^*^ time interaction effect on the FC of the right PI. **(A)** Denotes the altered FC in the patients compared with the HC. The cool color indicates decreased FC, and the hot color indicates increased FC. The maps are shown with probability scores between 80 and 100%. **(B)** Denotes the significant music intervention ^*^ time interaction that was observed in the PI-ACC FC and the PI-bilateral precentral FC. **(C)** The bar maps present the FC differences between-group and within-group. The data are expressed as the mean value + standard error. ^*^p < 0.05, ^**^p < 0.01. PM: probability map. The circles mark in **(B)**, which represents the positively modulated region through music intervention in patients in **(C)** (blue circle: ACC; black circle: left precentral; purple circle: right precentral).

The FC patterns of each seed were remarkably similar across the MTSZ, UMTSZ, and HC groups (Figure 2). Compared to the HCs, the patients demonstrated decreased FC between the three insular subregions and the orbital frontal cortex, as well as temporoparietal junction. In the left dAI and bilateral vAI networks, the patients exhibited decreased FC with the ACC. In addition, decreased FC was observed between the bilateral PI and the sensorimotor regions, as well as the occipital area in patients. While in the bilateral PI and vAI networks, the patients also demonstrated increased FC with the basal ganglia regions compared with the HCs (Figures 3A, 4A, 5A, Figure S1). In addition, compared to the UMTSZ group, the MTSZ patients demonstrated decreased correlations with the precentral and supplementary motor areas in the bilateral dAI networks (uncorrected *P* < 0.05; Figure S2). If the strict threshold (voxel level false discovery rate corrected *P* < 0.05) was used, no difference was observed between the MTSZ and UMTSZ groups at baseline.

### Music intervention effects in longitudinal changes of functional connectivity

After 1 month of music intervention, the repeated measures ANOVA analysis showed significant music intervention ^*^ time interaction effects on the FC between the left dAI and the ACC (Figure 3B, Table 3) in the patients. *Post-hoc* analysis revealed that the FC between the left dAI and the ACC did not show any significant differences between the MTSZ and UMTSZ groups at baseline but was found to be significantly increased in the MTSZ patients following music intervention (Figure 3C). Importantly, this increased connectivity was observed to decrease in patients compared with the HCs at baseline.

**Table 3:** Significant music intervention ^*^ time interaction on FC of subregion of insula through repeated measure ANOVA.

**Regions**	**BA**	**MNI coordinates**			**Peak *F*-score**	**Cluster voxels**
		**x**	**y**	**z**		
**LEFT dAI**						
Ins.L	BA 48	−38	−6	6	7.582	86
ROL.L	BA 48	−45	−5	10	5.474	
ACC.R	BA 24	5	24	26	11.756	56
ACC.L	BA 24	−2	21	26	7.263	
**LEFT PI**						
PreCG.L	BA 6	−57	5	27	13.421	118
ACC.R	BA 24	2	15	28	9.290	65
ACC.L	BA 24	−1	13	29	8.732	
**RIGHT PI**						
ACC.R	BA 24	2	12	30	11.528	165
ACC.L	BA 24	−2	13	29	11.344	
MCC.R	BA 24	1	9	33	10.173	
MCC.L	BA 24	−3	84	37	9.424	
PreCG.L	BA 6	−57	8	31	9.126	71
PreCG.R	BA 6	44	1	34	8.770	36

*BA, Brodmann area; dAI, dorsal anterior insula; PI, posterior insula; Ins, insula; ROL, rolandic operculum; PreCG, precentral gyrus; ACC, anterior cingulate cortex; MCC, middle cingulate cortex*.

Regions showing significant music intervention ^*^ time interaction effects on the FC of the left PI were located in the ACC and the left precentral gyrus (Figure 4B, Table 3), with non-significant lower values in the MTSZ group compared to the UMTSZ group at baseline and significant increases in the MTSZ group following music intervention (Figure 4C).

Significant music intervention ^*^ time interaction effects on the FC between the right PI and the ACC, left precentral, and right precentral gyrus were observed (Figure 5B, Table 3), with non-significant lower values in the MTSZ group compared to the UMTSZ group at baseline and significant increases in the MTSZ patients at the 1-month follow-up (Figure 5C). Finally, no significant main effects of time or group music intervention factors were observed in any regions. In the right dAI and bilateral vAI networks, no significant difference in the FC after music intervention was found.

**Figure 6:**
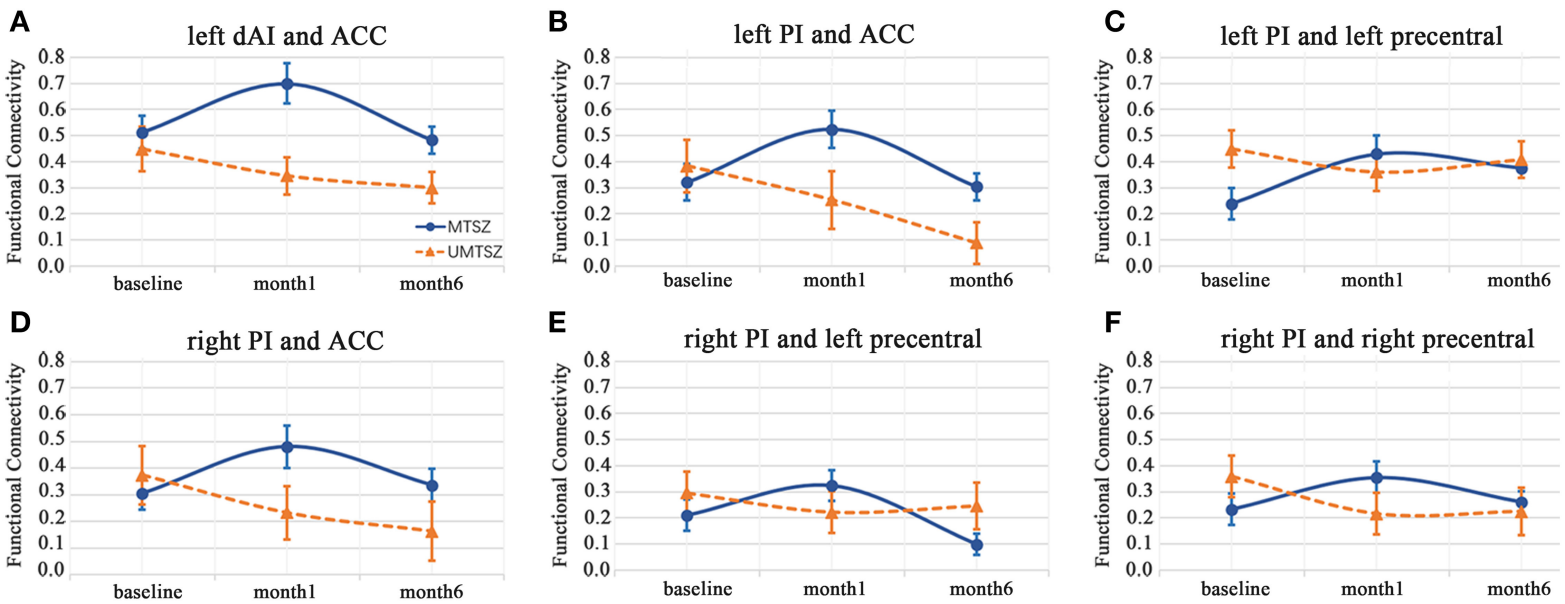
Six-month effects of music intervention versus no-music intervention on insular FC in the patients with schizophrenia. The data are expressed as the mean value ± standard error. **(A)** FC of left dAI and ACC; **(B)** FC of left PI and ACC; **(C)** FC of left PI and left precentral gyrus; **(D)** right PI and ACC; **(E)** right PI and left precentral gyrus; **(F)** right PI and right precentral gyrus.

The 6-month follow-up investigation is illustrated in Figure 6. The increased FC between the left insula (dAI and PI) and the ACC in response to the music intervention mentioned above had a significantly reduced alteration compared with the second scan (after 1-month of music intervention). In a word, a diminished effect of music intervention was observed after 6 months of music intervention.

### Prediction of music intervention effects by validation analyses

**Figure 7:**
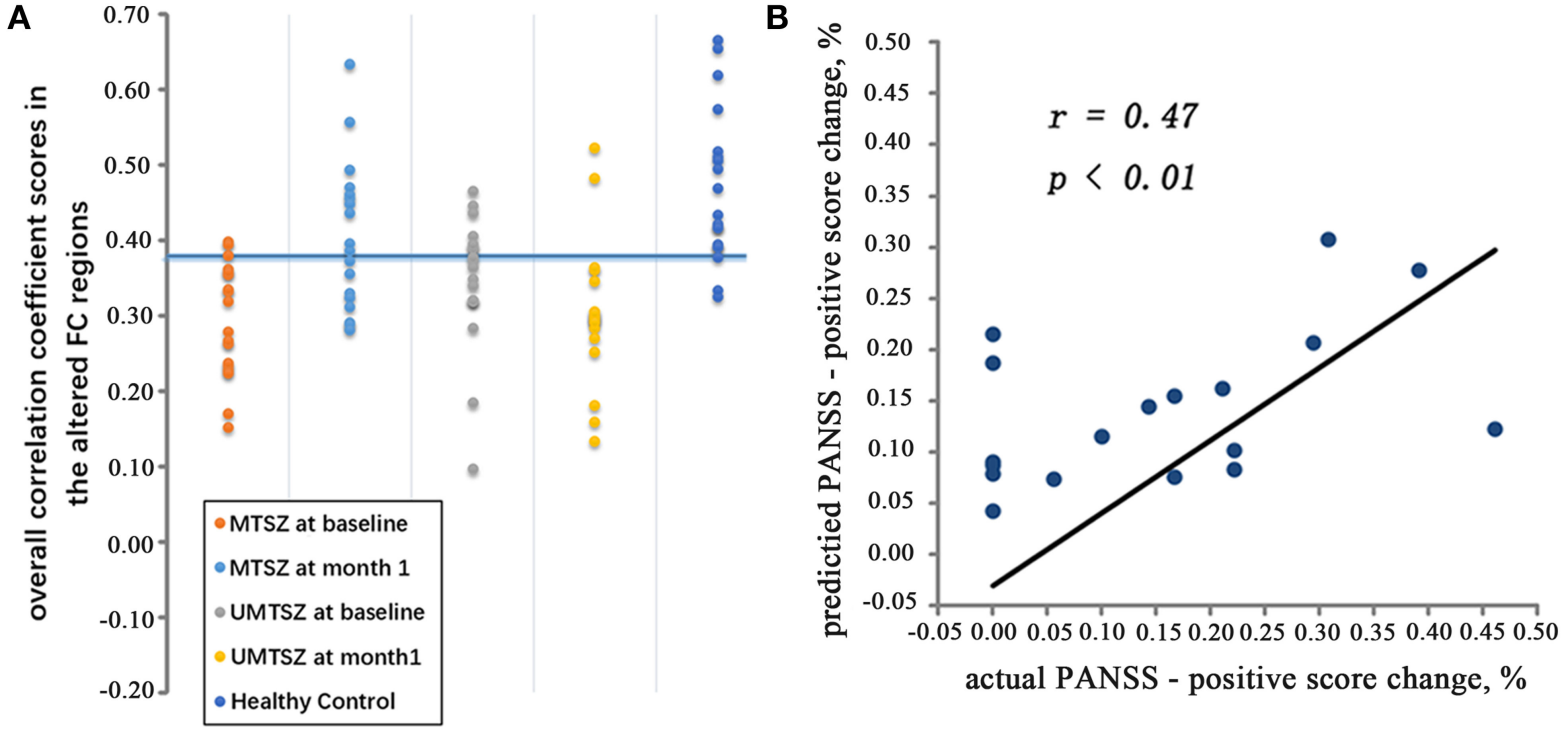
The pattern classification results. **(A)** Represents the validation result through the machine learning analysis. In the training cohort, the MTSZ at baseline and the MTSZ at 1-month were included. The validation cohort consisted of the UMTSZ at baseline, the UMTSZ at 1-month and the HCs. The blue line indicates a threshold with an 83.3% specificity and a 61.1% sensitivity for differentiating the MTSZ at 1-month from the MTSZ at baseline in the training cohort. Fitting this threshold to the validation cohort provided the accuracy for classifying the UMTSZ at baseline, the UMTSZ at 1-month and the HCs. **(B)** Represents the prediction result based on the SVR. The scatter map shows a significant correlation between the predicted and true individual percentage of change in the PANSS-positive score.

The binary and linear predictions of music intervention by the FCs yielded an accuracy rates significantly above the level of chance (Figure 7A, Table 4). In the classification model inter-group validation analysis, the classifiers_MTSZ, based on the FCs from MTSZ group, had better accuracy than the classifiers_UMTSZ. In addition, through this validation analysis, the effect of music intervention had vanished in the MTSZ group at the 6-month follow-up (Figure S3, Table S2). Detailed information about the validation analyses of the follow-up visits can be found in section 3 of the Supplemental Information.

**Table 4:** Validation performance (%) of classifiers based on FCs of MTSZ or UMTSZ.

**Classifier**	**Intra-group validation (LOOCV)**		**Inter-group validation (patients)**		**Inter-group validation (HCs)**
	**Sensitivity, %^a^**	**Specificity, %^b^**	**Sensitivity, %^a^**	**Specificity, %^b^**	**Sensitivity, %^c^**
SVM_MTSZ	83.33 (15/18)	66.67 (12/18)	77.78 (14/18)	11.11 (2/18)	84.21 (16/19)
SVM_UMTSZ	44.44 (8/18)	33.33 (6/18)	27.78 (5/18)	38.89 (7/18)	5.26 (1/19)
PHA_MTSZ	83.33 (15/18)	61.11 (11/18)	66.67 (12/18)	11.11 (2/18)	84.21 (16/19)
PHA_UMTSZ	22.22 (4/18)	44.44 (8/18)	16.67 (3/18)	33.33 (6/18)	10.53 (2/19)

*SVM, Support vector machines; PHA, post-hoc analysis; MTSZ, music intervention schizophrenia; UMTSZ, no-music intervention schizophrenia; HC, healthy control; LOOCV, leave one out cross validation*.

a *Sensitivity (true-positive rate) depicts the proportion at level without therapeutic effect (baseline) who are correctly identified in the inter-group validation (patients)*.

b *Specificity (true-negative rate) depicts the proportion at level with therapeutic effect (1-month) who are correctly identified in the inter-group validation (patients)*.

c *Sensitivity depicts the proportion of inter-group (HC) for classifying them as the state after intervention. Because there are two states: before(baseline) and after(1-month) intervention for any classifier, HC should be classified into after intervention state rather than before intervention state*.

Furthermore, the SVR results, which were based on the FC between the left dAI and the ACC in the MTSZ at baseline, were significantly associated with the actual percentage changes in the PANSS-positive scores of the MTSZ group (*r* = 0.47, *p* < 0.01; Figure 7B). The mean absolute error between the predicted percentage of change in the PANSS-positive score and the actual score was 8.37%.

## Discussion

To the best of our knowledge, this study is the first to assess the effect of long-term music intervention on the insular neural circuit in patients with schizophrenia. Consistent with previous studies, the patients with schizophrenia illustrated the dysfunctional insular connectivity in this study. After listening to Mozart's sonata music, we observed the positive improvement effect on the abnormally lower insular FCs (within the dAI and PI networks) using correlation and pattern classification analyses. Furthermore, the principal findings of the SVR analysis indicated that the FC of the left dAI with the ACC at baseline could predict the improvement in the psychiatric symptoms in the patients who received music intervention. However, at the 6-month follow-up investigation demonstrated the effect of music listening had vanished.

In patients with schizophrenia, abnormal external stimulus responses are central to its psychopathology (Morrison et al., 1988), weakening social cognition and regulation of emotion, which is associated with poor social functioning (Couture et al., 2006). Structural and functional deficits in the insula have been implicated in patients with schizophrenia for disturbed responses processing (Shepherd et al., 2012). The dAI plays a crucial role in the SN, which has extensive involvement in the detection and processing of salient events (Chang et al., 2013), in addition to its relation to marking objects that require further processing by integrating external stimuli with internal homeostatic contexts (Nomi et al., 2016). In schizophrenia, abnormal functioning of the anterior insula is involved in hallucinations (Wylie and Tregellas, 2010). Dysfunction of the SN has been observed in schizophrenia between the bilateral anterior insula and several nodes of the SN, which has been related to cognitive dysfunction (White et al., 2010). Similar to previous findings, at baseline, we observed decreased FC between the dAI and the ACC in schizophrenia. More importantly, in the MTSZ group, after listening to Mozart's sonata music, we found that long-term music intervention could positively improve the abnormally low FC between the dAI and the ACC. Extending these results here, in the MTSZ group, the SVR analysis provided a continuous prediction of positive symptom remission based on the baseline FC between the dAI and the ACC. Music listening can evoke emotional feelings, such as peacefulness, fear and joy (Sloboda et al., 2001). These effects could be related to music's ability to alter brain functional networks that are associated with the processing of external emotional stimuli (Brattico et al., 2013; Zatorre and Salimpoor, 2013). The dAI and the ACC, which are joined by interacting structural connections, are specialized for anticipation and evaluation of external stimuli (Lovero et al., 2009). These results indicated that the SN may be an important network for music intervention. Increased functional integration within the SN had a positive improvement effect on the abnormally low FC in the SN and might positively regulate the assignment of “salience events” in schizophrenia. This phenomenon might improve stimulus response and cognitive function in patients.

Of no less importance, schizophrenia is a severe mental disorder that is associated with derogated sensorimotor processing (Javitt, 2009). Neuroimaging studies have shown decreased connectivity in the sensorimotor functional networks in patients with schizophrenia (Walther and Strik, 2012; Chen et al., 2015). Furthermore, regions of the sensorimotor cortex have displayed widespread abnormal FC with higher-order regions in patients with schizophrenia (Kaufmann et al., 2015). Thus, it has been hypothesized that targeting the functional disintegration in sensorimotor processing domain might enhance our understanding of schizophrenia pathophysiology (Javitt, 2009). Specifically, the posterior insula is involved in sensorimotor processing (Stephani et al., 2011). It has been ascribed an integrative role, linking information from diverse sensorimotor functional regions and playing an important role in sensorimotor processing (Nieuwenhuys, 2012). In the current study, we observed decreased FC between the PI and sensorimotor regions in schizophrenia at baseline. After listening to Mozart's sonata music, we found increased FC between the bilateral PI and sensorimotor regions of the MTSZ group, though there were no significant differences in the patients compared with controls at the baseline. Previous studies have indicated that music listening can clearly modify the sensorimotor processing of the body, e.g., temperature and galvanic skin responses (Blood and Zatorre, 2001; Trost et al., 2012; Li et al., 2014). Our previous study also revealed that FC was significantly increased in the multi-sensorimotor cortices of musicians (Luo et al., 2012). Additionally, music intervention might be a useful neurorehabilitation tool for patients with chronic stroke and could lead to functional network reorganization in the sensorimotor cortex (Rojo et al., 2011). Thus, in our study, the increased sensorimotor connectivity after listening to Mozart's sonata music might be a compensatory response to other abnormal connectivity in the sensorimotor network in patients. Altogether, these findings may indicate that the increased FCs of the PI network, as the positive regulatory effect of long-term music intervention, might increase sensorimotor processing in patients with schizophrenia.

The functional differentiation of the insular cortex has already been indicated by excellent recent studies. The insula is thought to play a role in the functional integration between different functional systems by integrating information from these diverse systems (Craig, 2004; Nieuwenhuys, 2012). The insula was reported to be involved in not only integrating cognitive tasks and emotion, as well as in sensation, but also processing of the reciprocal influence of emotion and interoception (Critchley, 2005; Cao et al., 2016). Importantly, the dAI, as the key node of the SN, is the critical functional hub in these processes. A posterior-to-anterior progression of increasingly complex representations in the insula provides a foundation for the sequential integration of interoceptive awareness with the external sensory environment (Craig, 2002, 2011). This processing could then create subjective feelings from the integration of intero- and exteroceptive sensory information, providing a basis for the “self” and identification of the boundaries between the “self” and others (Namkung et al., 2017) that is considered a core abnormality of schizophrenia. Importantly, a recent study reported that music intervention as an addition to standard care could help patients with schizophrenia to improve their global state, mental state and social functioning (Mössler et al., 2011). In the MTSZ group, after listening to Mozart's sonata music, we found that long-term music intervention could improve the FC between the PI and ACC, which is one important region in the SN. In patients, the increased PI-SN FC might enhance the integration between the interoceptive and exteroceptive systems and improve both subjective feelings and the functioning between sensorimotor processing and response to an external stimulus. Consistent with this speculation, after music intervention in the MTSZ patients, we found remissions in the psychiatric symptoms, reflecting the effects of long-term music intervention. In addition, we found remission in the performance on the BVRT, which is a well-established neurodiagnostic instrument that assessed visuospatial perception and retention (Strauss et al., 2006) in the MTSZ group. Previous studies have indicated that the effects of music might not be durable in humans (Gardiner et al., 1996; Rauscher et al., 1997). In this study, the short-term effects of music intervention were also observed on insular subregion networks, psychiatric symptoms and BVRT performance. The abovementioned findings might reflect that music listening could temporarily normalize the reciprocal connections between the interoceptive and exteroceptive systems, then could relieve the psychiatric symptoms and behavior in patients with schizophrenia.

## Limitation

In this study, there were several limitations. First, the ideal design of our study, which included two groups of non-medically treated patients with schizophrenia, is ethically questionable for patients. Second, a single musical piece was selected (Mozart's sonata K. 448) in our study. The special effects of Mozart's music might be observed in patients. Further studies should investigate the effects of other types of music, such as general, familiar, and preferred music, in patients with schizophrenia. This future research might be a better way to understand the effect of different types of music on patients as well as to investigate whether these effects are similar to those obtained from Mozart music. Additionally, these additional results might help us to comprehend the mechanism behind the effect of music on the human brain. Third, our work only focused on the difference in the insular subregion networks. It is necessary to assess whether there are changes in other brain regions as well. The progressive effect of music intervention at the whole-brain level will be considered in future studies. Final, the results of this study require replication in larger samples; we believe that the present study advances the knowledge about the effect of music listening in patients with schizophrenia on the neural system, suggesting that the insular network, as the key core region in the salience and sensorimotor networks, may be an important target for music intervention, as well as improving the symptoms and behavior in patients with schizophrenia.

In conclusion, the human central nervous system continuously monitors the body's exterior environments through the SN. After music listening, increasing representations in the human insula could provide a foundation for the sequential integration of subjective feelings and motivational responses to external stimuli in patients. In addition, we found that the classification model of the MTSZ group had better accuracy in the classification analysis, which was caused by the normalized insular functional networks. The abovementioned findings might reflect that music intervention could normalize the salience and sensorimotor networks, as well as the relationship between these networks. This finding yields a significant remission in positive symptoms and behavior in response to music listening in patients with schizophrenia.

## Author contributions

HH, MY, CL, BB, and DY had made a substantial contribution to the conception and design the experiment and drafting and revising the article, then they gave final approval of the version to be published; YX and JS had made a substantial contribution to the analysis and interpretation of the data, and revising the article critically, and then he gave final approval of the version to be published; MD, XC, YL, DY, and CL had made a substantial contribution to the acquisition and interpretation of the data, then they gave final approval of the version to be published.

### Conflict of interest statement

The authors declare that the research was conducted in the absence of any commercial or financial relationships that could be construed as a potential conflict of interest.

## References

[B1] Baumgartner T. Lutz K. Schmidt C. F. Jäncke L. (2006). The emotional power of music: how music enhances the feeling of affective pictures. Brain Res. 1075, 151–164. 10.1016/j.brainres.2005.12.065 16458860

[B2] Blood A. J. Zatorre R. J. (2001). Intensely pleasurable responses to music correlate with activity in brain regions implicated in reward and emotion. Proc. Natl. Acad. Sci. U.S.A. 98, 11818–11823. 10.1073/pnas.191355898 11573015 PMC58814

[B3] Brattico E. Bogert B. Jacobsen T. (2013). Toward a neural chronometry for the aesthetic experience of music. Front. Psychol. 4:206. 10.3389/fpsyg.2013.00206 23641223 PMC3640187

[B4] Bruscia K. E. (1998). Defining Music Therapy. Spring House Books.

[B5] Cao W. Cao X. Hou C. Li T. Cheng Y. Jiang L.. (2016). Effects of cognitive training on resting-state functional connectivity of default mode, salience, and central executive networks. Front. Aging Neurosci. 8:70. 10.3389/fnagi.2016.00070 27148042 PMC4828428

[B6] Chan Y. M. Lee P. W. Ng T. Y. Ngan H. Y. Wong L. C. (2003). The use of music to reduce anxiety for patients undergoing colposcopy: a randomized trial. Gynecol. Oncol. 91, 213–217. 10.1016/S0090-8258(03)00412-8 14529684

[B7] Chang C. C. Lin C. J. (2011). LIBSVM: a library for support vector machines. ACM Trans. Intell. Syst. Technol. 2:27 10.1145/1961189.1961199

[B8] Chang L. J. Yarkoni T. Khaw M. W. Sanfey A. G. (2013). Decoding the role of the insula in human cognition: functional parcellation and large-scale reverse inference. Cereb. Cortex 23, 739–749. 10.1093/cercor/bhs065 22437053 PMC3563343

[B9] Chen X. Duan M. Xie Q. Lai Y. Dong L. Cao W.. (2015). Functional disconnection between the visual cortex and the sensorimotor cortex suggests a potential mechanism for self-disorder in schizophrenia. Schizophr. Res. 166, 151–157. 10.1016/j.schres.2015.06.014 26143483

[B10] Coppola G. Toro A. Operto F. F. Ferrarioli G. Pisano S. Viggiano A.. (2015). Mozart's music in children with drug-refractory epileptic encephalopathies. Epilepsy Behav. 50, 18–22. 10.1016/j.yebeh.2015.05.038 26093514

[B11] Couture S. M. Penn D. L. Roberts D. L. (2006). The functional significance of social cognition in schizophrenia: a review. Schizophr. Bull. 32(Suppl. 1), S44–S63. 10.1093/schbul/sbl029 16916889 PMC2632537

[B12] Craig A. D. (2002). How do you feel? Interoception: the sense of the physiological condition of the body. Nat. Rev. Neurosci. 3, 655–666. 10.1038/nrn894 12154366

[B13] Craig A. D. (2004). Human feelings: why are some more aware than others? Trends Cogn. Sci. 8, 239–241. 10.1016/j.tics.2004.04.004 15165543

[B14] Craig A. D. (2011). Significance of the insula for the evolution of human awareness of feelings from the body. Ann. N.Y. Acad. Sci. 1225, 72–82. 10.1111/j.1749-6632.2011.05990.x 21534994

[B15] Critchley H. D. (2005). Neural mechanisms of autonomic, affective, and cognitive integration. J. Comp. Neurol. 493, 154–166. 10.1002/cne.20749 16254997

[B16] Damasio A. (2001). Fundamental feelings. Nature 413:781. 10.1038/35101669 11677584

[B17] Deen B. Pitskel N. B. Pelphrey K. A. (2011). Three systems of insular functional connectivity identified with cluster analysis. Cereb. Cortex 21, 1498–1506. 10.1093/cercor/bhq186 21097516 PMC3116731

[B18] Dong D. Wang Y. Chang X. Luo C. Yao D. (2017). Dysfunction of large-scale brain networks in schizophrenia: a meta-analysis of resting-state functional connectivity. Schizophr. Bull. [Epub ahead of print]. 10.1093/schbul/sbx034 28338943 PMC5767956

[B19] Forman S. D. Cohen J. D. Fitzgerald M. Eddy W. F. Mintun M. A. Noll D. C. (1995). Improved assessment of significant activation in functional magnetic resonance imaging (fMRI): use of a cluster-size threshold. Magn. Reson. Med. 33, 636–647. 10.1002/mrm.1910330508 7596267

[B20] Fornito A. Zalesky A. Pantelis C. Bullmore E. T. (2012). Schizophrenia, neuroimaging and connectomics. Neuroimage 62, 2296–2314. 10.1016/j.neuroimage.2011.12.090 22387165

[B21] Foster N. A. Valentine E. R. (2001). The effect of auditory stimulation on autobiographical recall in dementia. Exp. Aging Res. 27, 215–228. 10.1080/036107301300208664 11441644

[B22] Gardiner M. F. Fox A. Knowles F. Jeffrey D. (1996). Learning improved by arts training. Nature 381:284. 10.1038/381284a0 8692266

[B23] Glicksohn J. Cohen Y. (2000). Can music alleviate cognitive dysfunction in schizophrenia? Psychopathology 33, 43–47. 10.1159/000029118 10601827

[B24] Gold C. Solli H. P. Krüger V. Lie S. A. (2009). Dose-response relationship in music therapy for people with serious mental disorders: systematic review and meta-analysis. Clin. Psychol. Rev. 29, 193–207. 10.1016/j.cpr.2009.01.001 19269725

[B25] Guétin S. Portet F. Picot M. Pommié C. Messaoudi M. Djabelkir L.. (2009). Effect of music therapy on anxiety and depression in patients with Alzheimer's type dementia: randomised, controlled study. Dement. Geriatr. Cogn. Disord. 28, 36–46. 10.1159/000229024 19628939

[B26] Habibi A. Damasio A. (2014). Music, feelings, and the human brain. Psychomusicol. Music Mind Brain 24, 92–102. 10.1037/pmu0000033

[B27] Hayashi N. Tanabe Y. Nakagawa S. Noguchi M. Iwata C. Koubuchi Y.. (2002). Effects of group musical therapy on inpatients with chronic psychoses: a controlled study. Psychiatry Clin. Neurosci. 56, 187–193. 10.1046/j.1440-1819.2002.00953.x 11952923

[B28] Javitt D. C. (2009). Sensory processing in schizophrenia: neither simple nor intact. Schizophr. Bull. 35, 1059–1064. 10.1093/schbul/sbp110 19833806 PMC2762632

[B29] Jiang Y. Duan M. Chen X. Chang X. He H. Li Y.. (2017). Common and distinct dysfunctional patterns contribute to triple network model in schizophrenia and depression: a preliminary study. Prog. Neuropsychopharmacol. Biol. Psychiatry 79(Pt B), 302–310. 10.1016/j.pnpbp.2017.07.007 28705767

[B30] Kaufman A. S. (2001). WAIS-III IQs, Horn's theory, and generational changes from young adulthood to old age. Intelligence 29, 131–167. 10.1016/S0160-2896(00)00046-5

[B31] Kaufmann T. Skåtun K. C. Alnæs D. Doan N. T. Duff E. P. Tønnesen S.. (2015). Disintegration of sensorimotor brain networks in schizophrenia. Schizophr. Bull. 41, 1326–1335. 10.1093/schbul/sbv060 25943122 PMC4601711

[B32] Kleber B. Friberg A. Zeitouni A. Zatorre R. (2017). Experience-dependent modulation of right anterior insula and sensorimotor regions as a function of noise-masked auditory feedback in singers and nonsingers. Neuroimage 147, 97–110. 10.1016/j.neuroimage.2016.11.059 27916664

[B33] Koelsch S. Fritz T. V Cramon DY. Müller K. Friederici A. D. (2006). Investigating emotion with music: an fMRI study. Hum. Brain Mapp. 27, 239–250. 10.1002/hbm.20180 16078183 PMC6871371

[B34] Kurth F. Zilles K. Fox P. T. Laird A. R. Eickhoff S. B. (2010). A link between the systems: functional differentiation and integration within the human insula revealed by meta-analysis. Brain Struct. Funct. 214, 519–534. 10.1007/s00429-010-0255-z 20512376 PMC4801482

[B35] Li G. He H. Huang M. Zhang X. Lu J. Lai Y.. (2015). Identifying enhanced cortico-basal ganglia loops associated with prolonged dance training. Sci. Rep. 5:10271. 10.1038/srep10271 26035693 PMC4649913

[B36] Li J. Luo C. Peng Y. Xie Q. Gong J. Dong L.. (2014). Probabilistic diffusion tractography reveals improvement of structural network in musicians. PLoS ONE 9:e105508. 10.1371/journal.pone.0105508 25157896 PMC4144874

[B37] Liang X. Zou Q. He Y. Yang Y. (2013). Coupling of functional connectivity and regional cerebral blood flow reveals a physiological basis for network hubs of the human brain. Proc. Natl. Acad. Sci. U.S.A. 110, 1929–1934. 10.1073/pnas.1214900110 23319644 PMC3562840

[B38] Linnman C. Coombs G. III. Goff D. C. Holt D. J. (2013). Lack of insula reactivity to aversive stimuli in schizophrenia. Schizophr. Res. 143, 150–157. 10.1016/j.schres.2012.10.038 23201307 PMC3540134

[B39] Lovero K. L. Simmons A. N. Aron J. L. Paulus M. P. (2009). Anterior insular cortex anticipates impending stimulus significance. Neuroimage 45, 976–983. 10.1016/j.neuroimage.2008.12.070 19280711 PMC3758834

[B40] Lu S. F. Lo C. H. Sung H. C. Hsieh T. C. Yu S. C. Chang S. C. (2013). Effects of group music intervention on psychiatric symptoms and depression in patient with schizophrenia. Complement. Ther. Med. 21, 682–688. 10.1016/j.ctim.2013.09.002 24280478

[B41] Luo C. Guo Z. W. Lai Y. X. Liao W. Liu Q. Kendrick K. M.. (2012). Musical training induces functional plasticity in perceptual and motor networks: insights from resting-state FMRI. PLoS ONE 7:e36568. 10.1371/journal.pone.0036568 22586478 PMC3346725

[B42] Luo C. Tu S. Peng Y. Gao S. Li J. Dong L.. (2014). Long-term effects of musical training and functional plasticity in salience system. Neural Plast. 2014:180138. 10.1155/2014/180138 25478236 PMC4247966

[B43] Menon V. Uddin L. Q. (2010). Saliency, switching, attention and control: a network model of insula function. Brain Struct. Funct. 214, 655–667. 10.1007/s00429-010-0262-0 20512370 PMC2899886

[B44] Mitchell R. L. Elliott R. Barry M. Cruttenden A. Woodruff P. W. (2004). Neural response to emotional prosody in schizophrenia and in bipolar affective disorder. Br. J. Psychiatry 184, 223–230. 10.1192/bjp.184.3.223 14990520

[B45] Moran L. V. Tagamets M. A. Sampath H. O'Donnell A. Stein E. A. Kochunov P.. (2013). Disruption of anterior insula modulation of large-scale brain networks in schizophrenia. Biol. Psychiatry 74, 467–474. 10.1016/j.biopsych.2013.02.029 23623456 PMC3735654

[B46] Morrison R. L. Bellack A. S. Mueser K. T. (1988). Deficits in facial-affect recognition and schizophrenia. Schizophr. Bull. 14, 67–83. 10.1093/schbul/14.1.67 3291095

[B47] Mössler K. Chen X. Heldal T. O. Gold C. (2011). Music therapy for people with schizophrenia and schizophrenia-like disorders. Cochrane Database Syst. Rev. CD004025 10.1002/14651858.CD004025.pub3 22161383

[B48] Namkung H. Kim S. H. Sawa A. (2017). The insula: an underestimated brain area in clinical neuroscience, psychiatry, and neurology. Trends Neurosci. 40, 200–207. 10.1016/j.tins.2017.02.002 28314446 PMC5538352

[B49] Nieuwenhuys R. (2012). The insular cortex: a review. Prog. Brain Res. 195, 123–163. 10.1016/B978-0-444-53860-4.00007-6 22230626

[B50] Nomi J. S. Farrant K. Damaraju E. Rachakonda S. Calhoun V. D. Uddin L. Q. (2016). Dynamic functional network connectivity reveals unique and overlapping profiles of insula subdivisions. Hum. Brain Mapp. 37, 1770–1787. 10.1002/hbm.23135 26880689 PMC4837017

[B51] Palaniyappan L. Mallikarjun P. Joseph V. Liddle P. F. (2011). Appreciating symptoms and deficits in schizophrenia: right posterior insula and poor insight. Prog. Neuropsychopharmacol. Biol. Psychiatry 35, 523–527. 10.1016/j.pnpbp.2010.12.008 21182887

[B52] Pavlicevic M. Trevarthen C. Duncan J. (1994). Improvisational music therapy and the rehabilitation of persons suffering from chronic schizophrenia. J. Music Ther. 53, 2955–2959. 10.1093/jmt/31.2.86

[B53] Power J. D. Barnes K. A. Snyder A. Z. Schlaggar B. L. Petersen S. E. (2012). Spurious but systematic correlations in functional connectivity MRI networks arise from subject motion. Neuroimage 59, 2142–2154. 10.1016/j.neuroimage.2011.10.018 22019881 PMC3254728

[B54] Rauscher F. H. Shaw G. L. Ky K. N. (1993). Music and spatial task performance. Nature 365:611. 10.1038/365611a0 8413624

[B55] Rauscher F. H. Shaw G. L. Levine L. J. Wright E. L. Dennis W. R. Newcomb R. L. (1997). Music training causes long-term enhancement of preschool children's spatial-temporal reasoning. Neurol. Res. 19, 2–8. 10.1080/01616412.1997.11740765 9090630

[B56] Rector N. A. Beck A. T. (2001). Cognitive behavioral therapy for schizophrenia: an empirical review. J. Nerv. Ment. Dis. 189, 278–287. 10.1097/00005053-200105000-00002 11379970

[B57] Rojo N. Amengual J. Juncadella M. Rubio F. Camara E. Marco-Pallares J.. (2011). Music-supported therapy induces plasticity in the sensorimotor cortex in chronic stroke: a single-case study using multimodal imaging (fMRI-TMS). Brain Inj. 25, 787–793. 10.3109/02699052.2011.576305 21561296

[B58] Rolvsjord R. (2001). Sophie learns to play her songs of tears. Nordic J. Music Ther. 10, 77–85. 10.1080/08098130109478020

[B59] Shepherd A. M. Matheson S. L. Laurens K. R. Carr V. J. Green M. J. (2012). Systematic meta-analysis of insula volume in schizophrenia. Biol. Psychiatry 72, 775–784. 10.1016/j.biopsych.2012.04.020 22621997

[B60] Singer T. Critchley H. D. Preuschoff K. (2009). A common role of insula in feelings, empathy and uncertainty. Trends Cogn. Sci. 13, 334–340. 10.1016/j.tics.2009.05.001 19643659

[B61] Sloboda J. A. O'Neill S. A. Ivaldi A. (2001). Functions of music in everyday life: an exploratory study using the experience sampling method. Music. Sci. 5, 9–32. 10.1177/102986490100500102

[B62] Solli H. P. (2008). “Shut up and play!” Improvisational use of popular music for a man with schizophrenia. Nordic J. Music Ther. 17, 67–77. 10.1080/08098130809478197

[B63] Stephani C. Fernandez-Baca Vaca G. Maciunas R. Koubeissi M. Lüders H. O. (2011). Functional neuroanatomy of the insular lobe. Brain Struct. Funct. 216, 137–149. 10.1007/s00429-010-0296-3 21153903 PMC3097350

[B64] Strauss E. Sherman E. M. Spreen O. (2006). A Compendium of Neuropsychological Tests: Administration, Norms, and Commentary. Oxford: Oxford University Press.

[B65] Talwar N. Crawford M. J. Maratos A. Nur U. McDermott O. Procter S. (2006). Music therapy for in-patients with schizophrenia: exploratory randomised controlled trial. Br. J. Psychiatry 189, 405–409. 10.1192/bjp.bp.105.015073 17077429

[B66] Tamkin A. S. Kunce J. T. (1985). A comparison of three neuropsychological tests: the Weigl, Hooper, and Benton. J. Clin. Psychol. 41, 660–664. 4044848 10.1002/1097-4679(198509)41:5<660::aid-jclp2270410512>3.0.co;2-b

[B67] Tang W. Yao X. Zheng Z. (1994). Rehabilitative effect of music therapy for residual schizophrenia. A one-month randomised controlled trial in Shanghai. Br. J. Psychiatry Suppl. 164, 38–44. 7946230

[B68] Trost W. Ethofer T. Zentner M. Vuilleumier P. (2012). Mapping aesthetic musical emotions in the brain. Cereb. Cortex 22, 2769–2783. 10.1093/cercor/bhr353 22178712 PMC3491764

[B69] Uddin L. Q. Kinnison J. Pessoa L. Anderson M. L. (2014). Beyond the tripartite cognition-emotion-interoception model of the human insular cortex. J. Cogn. Neurosci. 26, 16–27. 10.1162/jocn_a_00462 23937691 PMC4074004

[B70] Ulrich G. Houtmans T. Gold C. (2007). The additional therapeutic effect of group music therapy for schizophrenic patients: a randomized study. Acta Psychiatr. Scand. 116, 362–370. 10.1111/j.1600-0447.2007.01073.x 17919155

[B71] Walther S. Strik W. (2012). Motor symptoms and schizophrenia. Neuropsychobiology 66, 77–92. 10.1159/000339456 22814247

[B72] Waters F. A. Badcock J. C. (2010). First-rank symptoms in schizophrenia: reexamining mechanisms of self-recognition. Schizophr. Bull. 36, 510–517. 10.1093/schbul/sbn112 18753307 PMC2879682

[B73] Wechsler D. (1981). WAIS-R Manual: Wechsler Adult Intelligence Scale-Revised. New York, NY: Psychological Corporation.

[B74] White T. P. Joseph V. Francis S. T. Liddle P. F. (2010). Aberrant salience network (bilateral insula and anterior cingulate cortex) connectivity during information processing in schizophrenia. Schizophr. Res. 123, 105–115. 10.1016/j.schres.2010.07.020 20724114

[B75] Williams L. M. Das P. Liddell B. J. Olivieri G. Peduto A. S. David A. S.. (2007). Fronto-limbic and autonomic disjunctions to negative emotion distinguish schizophrenia subtypes. Psychiatry Res. 155, 29–44. 10.1016/j.pscychresns.2006.12.018 17398080

[B76] Wojtalik J. A. Smith M. J. Keshavan M. S. Eack S. M. (2017). A systematic and meta-analytic review of neural correlates of functional outcome in schizophrenia. Schizophr. Bull. 43, 1329–1347. 10.1093/schbul/sbx008 28204755 PMC5737663

[B77] Wylie K. P. Tregellas J. R. (2010). The role of the insula in schizophrenia. Schizophr. Res. 123, 93–104. 10.1016/j.schres.2010.08.027 20832997 PMC2957503

[B78] Xing Y. Xia Y. Kendrick K. Liu X. Wang M. Wu D.. (2016). Mozart, Mozart rhythm and retrograde Mozart effects: evidences from behaviours and neurobiology bases. Sci. Rep. 6:18744. 10.1038/srep18744 26795072 PMC4726287

[B79] Yang G. J. Murray J. D. Repovs G. Cole M. W. Savic A. Glasser M. F.. (2014). Altered global brain signal in schizophrenia. Proc. Natl. Acad. Sci. U.S.A. 111, 7438–7443. 10.1073/pnas.1405289111 24799682 PMC4034208

[B80] Yeo B. T. Krienen F. M. Eickhoff S. B. Yaakub S. N. Fox P. T. Buckner R. L.. (2014). Functional specialization and flexibility in human association cortex. Cereb. Cortex 25, 3654–3672. 10.1093/cercor/bhu217 25249407 PMC4598819

[B81] Zamorano A. M. Cifre I. Montoya P. Riquelme I. Kleber B. (2017). Insula-based networks in professional musicians: evidence for increased functional connectivity during resting state fMRI. Hum. Brain Mapp. 38, 4834–4849. 10.1002/hbm.23682 28737256 PMC6866802

[B82] Zatorre R. J. Salimpoor V. N. (2013). From perception to pleasure: music and its neural substrates. Proc. Natl. Acad. Sci. U.S.A. 110(Suppl. 2), 10430–10437. 10.1073/pnas.1301228110 23754373 PMC3690607

