# Supplementary material for: Music Intervention Leads to Increased Insular Connectivity and Improved Clinical Symptoms in Schizophrenia
Source: Front Neurosci. 2018 Jan 23;11:744. doi: 10.3389/fnins.2017.00744 (PMC5787137; doi:10.3389/fnins.2017.00744)
Supplement: Supplementary file 1 [file Presentation1.PDF]

Music intervention leads to increased insular connectivity and improved clinical symptoms in schizophrenia

Supplemental Information

Hui He[1#], Mi Yang[1, 2#], Mingjun Duan[1, 2], Xi Chen[1], Yang Xia[1], Junming Shao[1], Bharat B. Biswal[1], Cheng Luo[1*], Dezhong Yao[1*]

1, The Clinical Hospital of Chengdu Brain Science Institute, MOE Key Lab for Neuroinformation, University of Electronic Science and Technology of China, Chengdu, China

2, The Four People's Hospital of Chengdu, Chengdu, 610036, China

*Corresponding Authors: Cheng Luo, chengluo@uestc.edu.cn; tel: +86 28 83201018; Fax: +86 (0)28 83206972; Also to Dezhong Yao, dyao@uestc.edu.cn; +86 (0)28 8320 1018; fax: +86 28 8320 8238

HH and MY contributed to this work equally

Section 1. The medication dosage information of the patients

STable 1. The medication dosage information of the MTSZ and UMTSZ groups

Patient No.	MTSZ<br>Medication dosage in CPZ equivalents (mg)	UMTSZ<br>Medication dosage in CPZ equivalents (mg)
1	441.92	151.52
2	360.12	146.30
3	280.49	561.42
4	270.56	208.33
5	601.85	370.37
6	454.55	488.22
7	324.07	303.03
8	313.60	281.69
9	303.03	208.33
10	227.86	430.34
11	317.80	69.44
12	231.48	458.79
13	347.22	395.62
14	277.78	155.76
15	347.81	471.38
16	422.54	303.03
17	352.11	462.96
18	231.48	303.03

Abbreviations: MTSZ: music intervention schizophrenia; UMTSZ: no-music intervention schizophrenia; CPZ, chlorpromazine;

Section2. Functional connectivity abnormalities at baseline

We established baseline abnormalities between MTSZ and UMTSZ through two-sample t-test, accounting for the effects of gender, years of education, GM, and age, with an explicit mask from the union set of the one-sample t-test results of the two groups. Compared to UMTSZ, the altered rsFCs of MTSZ were present in SFigure 2 (uncorrected $p < 0.05$). For bilateral dAI the MTSZ patients revealed decreased correlations with precentral and supplementary motor area (SFigure 2). If the strict threshold (false discovery rate corrected $p < 0.05$) was used, no difference was observed between MTSZ and UMTSZ group at baseline. In addition, these regions were not observed significantly altered FC in the following repeat measured ANOVA analysis.

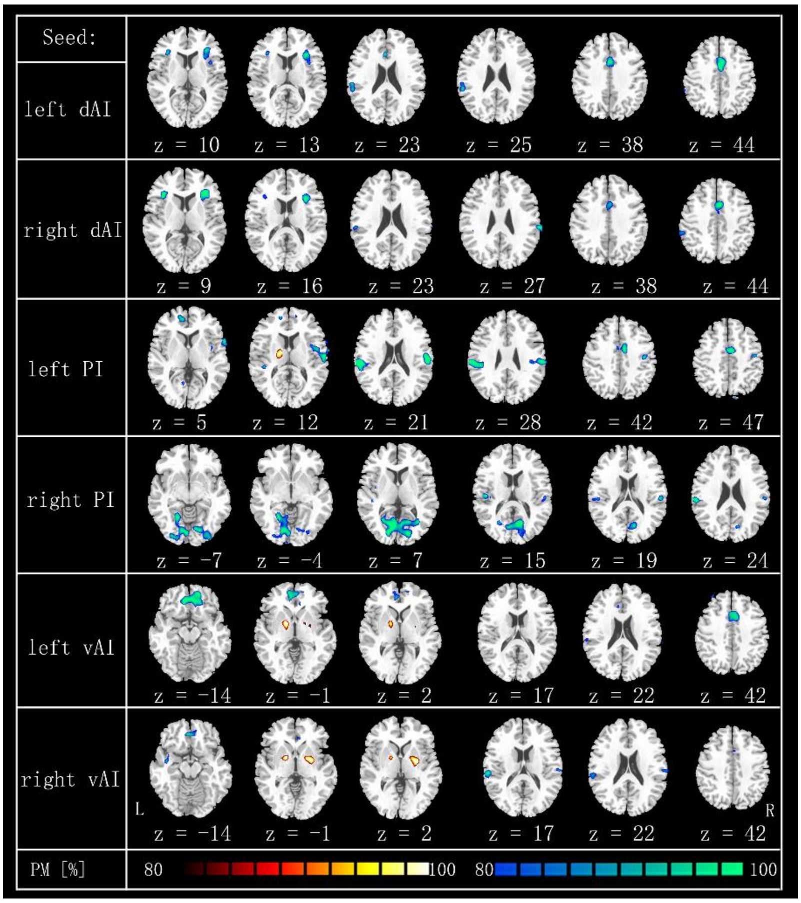

SFigure 1. Resting-state functional connection findings of each seed in the baseline. Compared to healthy controls, the altered FCs of patients with schizophrenia were displayed (the probability value > 0.8, $k = 23$ adjacent voxels). '*A* 'denotes the altered results of the left dAI FC network ('*B'*: right dAI; '*C'*: left PI; '*D'*: right PI; '*E'*: left vAI; '*F'*: right vAI). The cool color indicates decreased functional connections and hot color indicates increased functional connections, when patients with schizophrenia was compared to healthy controls. Abbreviations: PM: probability map.

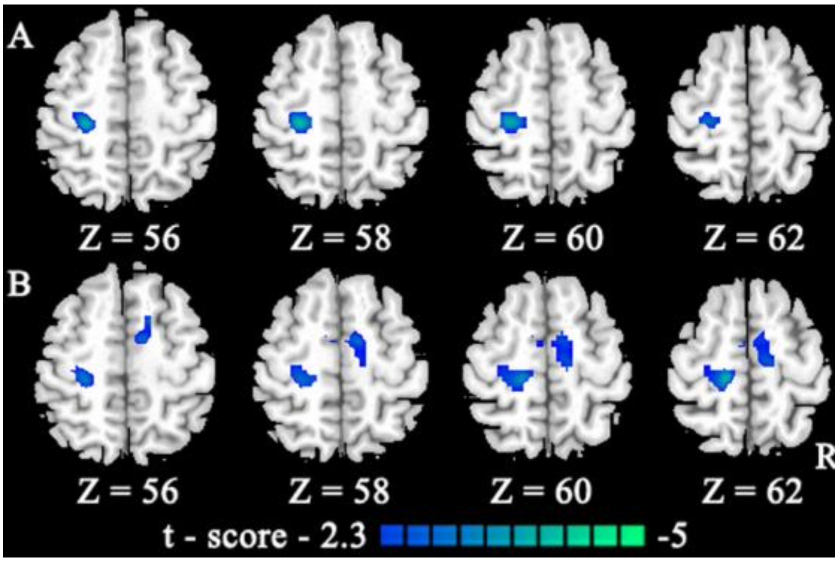

SFigure 2. Resting-state functional connection differences between MTSZ and UMTSZ in the baseline. Compared to UMTSZ, the altered FCs of MTSZ were displayed (uncorrected $p < 0.05$). '*A*' denotes the altered results of the left dAI FC network, and '*B'* represents right dAI). The cool color indicates decreased functional connections, when MTSZ was compared to UMTSZ. All of the maps are shown with t score between -2.3 and -5. In addition, these regions were not observed significantly altered FC in the following repeat measured ANOVA analysis.

Section 3. The validation results through machine learning analysis

In the intra-group validation analyses, we used the leave one out cross validation (LOOCV) strategy to predict the therapeutic effect in MTSZ and UMTSZ groups respectively. In the training step, each patient was designated the validation sample in turns while the remaining samples were used to train the classification model. The altered FCs, which resulted from the repeated measure ANOVA, was used as features. Then, in the validation step, we predict the therapeutic effect using the same feature. In the inter-group validation analyses, two classification models were built based on the data of MUSZ and UMTSZ respectively. The altered FCs, which resulted from the repeated measure ANOVA, was used as features. Then, two decision models were respectively used to predict the therapeutic effect in the validation groups (the other patient and HC groups).

First, a support vector machine (SVM) was applied. A linear kernel was used with default settings and the parameter C=1. The classification model of SVM_MTSZ was built based on data of MTSZ group, and SVM_UMTSZ model based on data of UMTSZ group. Then, two decision models were respectively used to predict the therapeutic effect of the validation subjects. Validation analysis was implemented using the SVM toolbox (Chang and Lin, 2011). The validation performances of two SVM classification models were adopted to evaluate the therapeutic effect.

Second, similar abovementioned steps, two post hoc analyses (PHA_MTSZ and PHA_UMTSZ) were also performed on the overall correlation coefficient (OCC) values. The post hoc analysis (PHA) was performed on the OCC values in the altered FC regions. The mean correlation coefficient was averaged participant-wise within a binary mask containing only significant clusters showed significant altered FC in six insular subregions FC networks respectively, which produced six values for each participant. Then, we got the overall correlation coefficient values through averaging the six values. The OCC values were obtained for the training groups (MTSZ or UMTSZ) and predict the therapeutic effect in validation subjects. Then, a receiver operating characteristics (ROC) curve was generated to identify the optimal threshold for classifying the training group (baseline and 1-month) based on the OCC values. The threshold identified in the ROC analysis was used to predict the therapeutic effect of the validation groups.

No significant association was observed between medication dosage and the pattern classification test margins ($p > 0.5$).

To further examine the effect of music therapy on insular networks, we did the follow-up visits with MRI scan after 6-month later, as the effect of music therapy would be vanished gradually after the period of treatment (Guetin et al., 2009). However, nine patients with schizophrenia (25%) were not recalled because of several reasons. Five patients with schizophrenia with excessive head motion were also excluded, so thirteen (13/18) UMTSZ and nine (9/18) MTSZ patients were included followed-up analysis. Then, the validation analysis was performed in MTSZ and UMTSZ groups at 6-month later respectively. Firstly, the same features were extracted from the MTSZ and UMTSZ group. Secondly, we predicted the label of followed-up visited patients through two original decision models

SVM and PHA respectively, which built by the data from baseline and 1-month later in two groups. High accuracy of without music intervention were observed in MTSZ and UMTSZ groups. Thus, vanished effect of music intervention was observed in MTSZ group at 6-month later (SFigure 3 and STable 2).

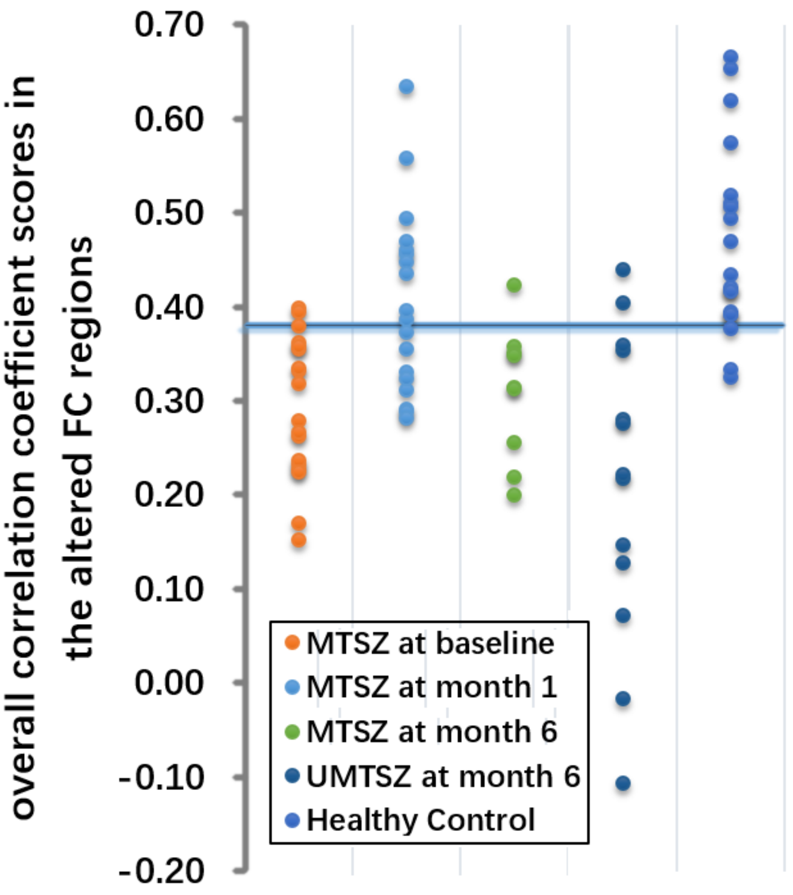

SFigure3. The validation results. This is the overall correlation coefficient scores in the altered FC regions through repeat measure ANOVA for the data of 6-month later. In the training cohort, MTSZ at baseline and MTSZ at 1-month were included. The validation cohort consists of MTSZ at 6-month, UMTSZ at 6-month and HC. The blue line indicates a threshold with 83.3% specificity and 61.1% sensitivity for differentiating MTSZ at 1-month from MTSZ at baseline in the training cohort. Fitting this threshold to the validation cohort provides the accuracy for classifying MTSZ at 6-month, UMTSZ at 6-month and HC.

STable 2. Validation performance (%) of classifiers based on FCs of MTSZ or UMTSZ

	Intra-group Validation (LOOCV)		Inter-group Validation (patients, 6-month later)	
Classifier	Sensitivity, % [a]	Specificity, % [b]	Specificity (MTSZ), % [c]	Specificity (UMTSZ), % [c]
SVM_MTSZ	83.33 (15/18)	66.67 (12/18)	11.11 (1/9)	7.69 (1/13)
SVM_UMTSZ	44.44 (8/18)	33.33 (6/18)	33.33 (3/9)	30.77 (4/13)
PHA_MTSZ	83.33 (15/18)	61.11 (11/18)	11.11 (1/9)	15.38 (2/13)
PHA_UMTSZ	22.22 (4/18)	44.44 (8/18)	11.11 (1/9)	15.38 (2/13)

Abbreviations: SVM: support vector machines; PHA: post hoc analysis; MTSZ: music intervention schizophrenia; UMTSZ: no-music intervention schizophrenia; HC: healthy control.

[a] Sensitivity (true-positive rate) depicts the proportion at level without music therapeutic effect (baseline) who are correctly identified.

[b] Specificity (true-negative rate) depicts the proportion at with music therapeutic effect (1-month later) who are correctly identified.

[c] Sensitivity depicts the proportion at level with music therapeutic effect (6-month later) who are correctly identified in patients.

Supplementary References:

Chang, C.C., and Lin, C.J. (2011). LIBSVM: A Library for Support Vector Machines. *Acm Transactions on Intelligent Systems and Technology* 2(3). doi: Artn 27
Doi 10.1145/1961189.1961199.

Guetin, S., Portet, F., Picot, M., Pommié, C., Messaoudi, M., Djabelkir, L., et al. (2009). Effect of music therapy on anxiety and depression in patients with Alzheimer’s type dementia: randomised, controlled study. *Dementia and geriatric cognitive disorders* 28(1)**,** 36-46.
